# Super-hydrophobic multilayer coatings with layer number tuned swapping in surface wettability and redox catalytic anti-corrosion application

**DOI:** 10.1038/s41598-017-04651-3

**Published:** 2017-06-30

**Authors:** Junaid Ali Syed, Shaochun Tang, Xiangkang Meng

**Affiliations:** National Laboratory of Solid State Microstructures, Collaborative Innovation Center of Advanced Microstructures, College of Engineering and Applied Sciences, and Institute of Materials Engineering, Nanjing University, Nanjing, Jiangsu People’s Republic of China

## Abstract

The wetting characteristic of a metal surface can be controlled by employing different coating materials and external stimuli, however, layer number (*n*) modulated surface swapping between hydrophobicity and hydrophilicity in a multilayer structure to achieve prolonged anti-corrosion ability was not taken into consideration. In this study, we proposed a layer-by-layer (LbL) spin assembled polyaniline-silica composite/tetramethylsilane functionalized silica nanoparticles (PSC/TMS-SiO_2_) coating with the combined effect of super-hydrophobicity and enhanced anti-corrosion ability. Interestingly, the hierarchical integration of two coating materials with inherently different surface roughness and energy in a multilayer structure allows the wetting feature to swap from hydrophobic to hydrophilic state by modulating *n* with decreasing hydrophilicity. The samples with odd *n* (TMS-SiO_2_ surface) are hydrophobic while the samples with even n (PSC surface) exhibits the hydrophilic character. The TMS-SiO_2_ content was optimized to achieve super-hydrophobic coating with significantly high water contact angle (CA) 153° ± 2° and small sliding angle (SA) 6° ± 2°. Beside its self-cleaning behavior, the electro-active PSC/TMS-SiO_2_ coating also exhibits remarkably enhanced corrosion resistance against aggressive media. The corrosion resistance of the coating was remained stable even after 240 h of exposure, this enhancement is attributed to super-hydrophobicity and anodic shift in corrosion potential.

## Introduction

Corrosion is a thermodynamically pivotal process which consumes 3% of the world gross domestic product (GDP) annually^1, 2^. This process would not only affect the world’s economy but also lead to harmful impact on industry and human societies. Among different alloys and metals, stainless steel (SS) and iron are the most extensively used material in industries due to its good mechanical strength^3, 4^; however, they are prone to corrosion due to their prominent electrochemical activity. It is well-known that corrosion is hard to prevent, hence it is strongly desired to decelerate the corrosion reactions and alter its kinetics by changing the mechanism involved in it^3, 5^. As an alloy, SS contains chromium in its composition which reacts with the atmospheric oxygen to form a passive oxide layer. This oxide layer retains the surface integrity of the SS, in spite of this chloride ions could penetrate through the porous oxide layer and initiates corrosion^6, 7^. However, treatment of SS with chromates is forbidden due to hazardous environmental concerns associated with its usage^8, 9^. Therefore, SS is not encouraged for applications where chloride containing environment is present unless it is protected with a particular coating. One of the common practices is the development of conductive polymeric (CP) coatings to protect metal from aggressive enviornment^10–12^. Among these, polyaniline (PANI) coating plays a vital role, due to its redox catalytic ability which contributes to the formation of an oxide layer on a metal surface, thus improves its corrosion resistance^13, 14^. On the other hand, CP based coatings have failed to protect metal for prolonged time due to its porous nature which allows the diffusion of some corrosive species, i.e., atmospheric oxygen, chloride ions and water^15, 16^. In contrast, the inorganic-organic hybrid coating with super-hydrophobic property would not only contribute to increase the corrosion resistance of the metal with excellent barrier ability but also introduce self-cleaning ability as well.

Over the past few years, super-hydrophobic surfaces inspired from highly functional and exceptional natural designs such as “lotus leaf” were attracting researchers to gain maximum monetary benefits^17, 18^. The super-hydrophobic surfaces exhibits high static contact angle (CA) greater than 150° and small sliding angle (SA) less than 10°^19^. The two important factors i.e., low surface energy material and surface roughness contributes to the biomimetic super-hydrophobic structure which assist researchers to develop artificial super-hydrophobic surfaces with modulated wettability^20, 21^. Furthermore, it is desirable to control the swapping of solid surface between the hydrophilic and hydrophobic states via external stimuli^22, 23^, which is important for many applications such as self-cleaning^24^, antifogging, oil/water separations^25^, anti-reflection^26^, and anti-corrosion^27–29^. For reversible surface wettability, the material should inherently have the ability to swap between hydrophobic and hydrophilic states as a function of external stimuli before any modification in surface roughness. However, a limited number of materials are available with this unique property and required specific fabrication method against different stimuli. Therefore, a general method is desired to fabricate materials with different wettability which can be controlled by an internal or external factor. This control on surface wetting characteristic would provide a controlled diffusion of electrolyte within the coating and hence one can tune the coating to protect the metal surface for prolonged time. Recently, few attempts have been made to impart super-hydrophobicity on metal surface for corrosion protection by employing electro-active polymer and epoxy coatings using nano-casting technique^30–32^. However, the nano-casting method involves complex steps and the fabricated coatings are not capable of surface wettability switch with lack of long-term protection ability. On the other hand, the layer-by-layer (LbL) assembly of polymers is one of the versatile approaches to modify the surface properties of the substrate^33^. The most facile strategy to increase the surface roughness of the LbL polymers is the addition of inorganic nanoparticles (NPs) followed by surface hydrophobization to obtain controlled wetting property^34^. The sum up is, LbL polymer coating without inorganic additives and lack of controllable water repellent ability not able to protect the metal surface against aggressive environment for long-term. Therefore, it is strongly desired to develop a defect free coating with the combined effect of super-hydrophobicity and CP redox catalytic ability for long term corrosion protection of SS by a facile coating technique which provides a control over surface wettability.

In our previous publications, we proposed a facile and robust layer-by-layer (LbL) method to develop polyelectrolyte multilayer coatings to achieve enhanced corrosion protection for SS with PANI redox catalytic ability^35^ and self-healing^36^. However, PANI based super-hydrophobic composite coatings with modulated surface wettability for anti-corrosion application were rarely reported^31, 37^. In this work, we demonstrate the fabrication of PSC/TMS-SiO_2_ super-hydrophobic LbL multilayer coatings with controlled wettability for prolonged corrosion protection of the SS by spin-assembly. Our proposed strategy has several advantages such as, the co-assembly of two materials PSC and TMS-SiO_2_ with different surface roughness and energy in a multilayer fashion would allow layer number (*n*) to act as a switch to modulate surface wettability; the change in hydrophobicity to hydrophilicity can be controlled over a wide range; *n* can be used to control the swapping and this control is tunable to achieve self-cleaning ability; the super-hydrophobic coating with redox catalytic ability twofold the corrosion performance of metals for a long period of time due to enhanced barrier ability.

## Results and Discussion

**Figure 1:**
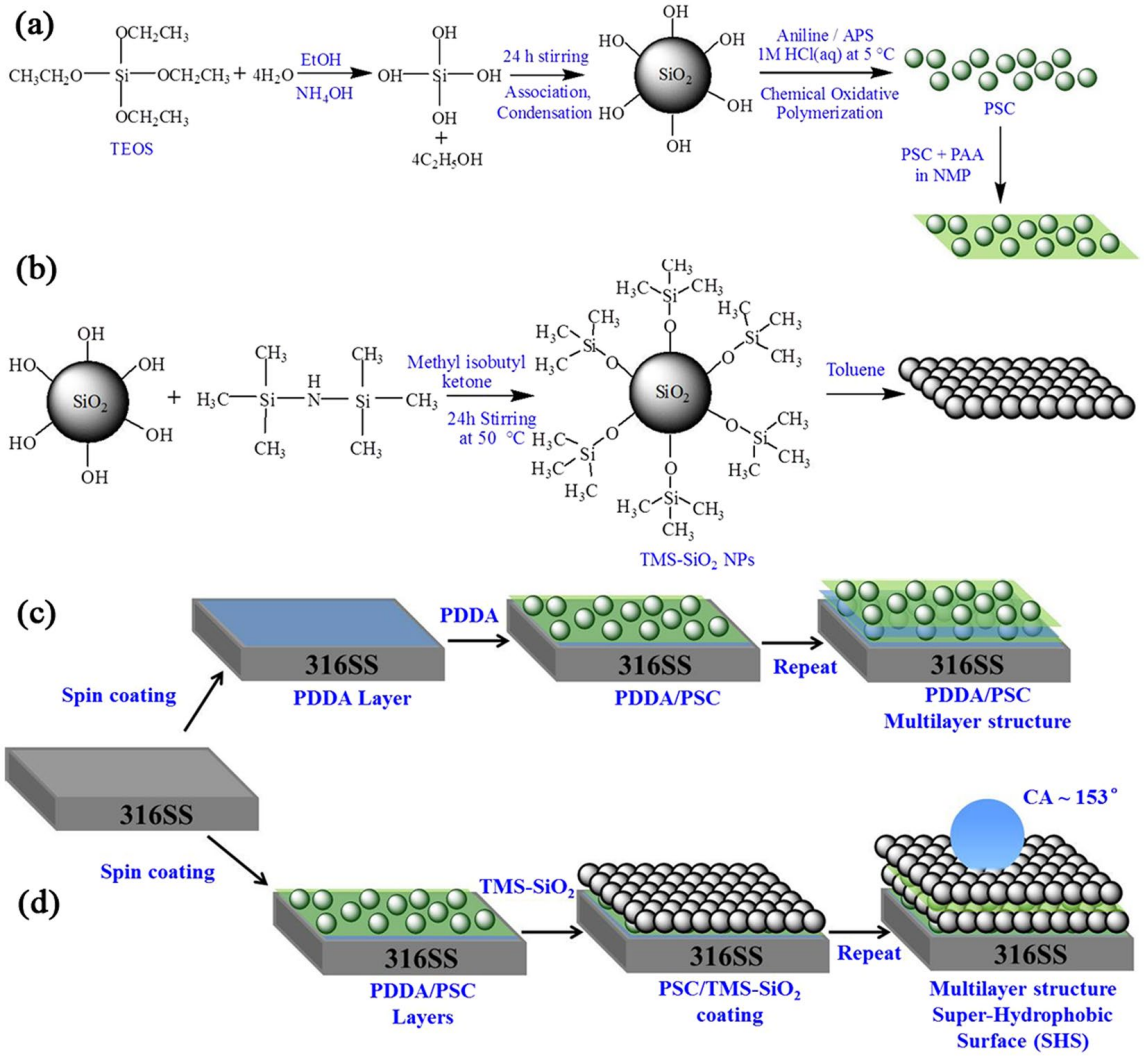
The schematic shows the synthesis of (**a**) PSC composite and preparation of its coating solution, (**b**) surface modification of silica to obtain TMS-SiO_2_ NPs. Fabrication of (**c**) PDDA/PSC and (**d**) PSC/TMS-SiO_2_ multilayers on 316SS by spin assembly.

Figure 1 displays the schematics for the synthesis of PSC composite and modification of silica particles into TMS-SiO_2_ spheres with their respective coating solution and the fabrication process of PDDA/PSC and PSC/TMS-SiO_2_ multilayers.

### Characterization of Coating Material

The formation of PANI and its PSC composite was confirmed by the FTIR spectra in Fig. S1a and S1b (Supplementary Information). The peaks at 1346 and 1615 cm^−1^ were assigned to C=C stretching of benzenoid and quinoid rings^38^. The bands at 1163 and 823 cm^−1^ in Fig. S1a were attributed to the C-H bending vibration and primary amines, respectively^18^. The peak at 1099 cm^−1^ was attributed to the symmetric stretching of Si-O-Si in Fig. S1b, S1c and S1d. The presence of bands related to PANI and SiO_2_ in PSC spectra implies the formation of the PSC composite (Fig. S1b) and Fig. S1e shows the surface modification of SiO_2_ particles with HDMS. Both the spectra in Fig. S1c and S1d shows a broad band around 3435 cm^−1^ and the weak band at 1630 cm^−1^ attributed to the presence of little water in the sample^39^. In Fig. S1d, the peaks at 2835 and 2780 cm^−1^ corresponds to asymmetric and symmetric CH stretching (magnified inset of Fig. S1c and S1d), these peaks confirms the surface modification of SiO_2_ particles with TMS^40, 41^. The presence of Si(CH_3_)_3_ rocking vibration was confirmed by the weak peak at 785 cm^−1^ ^40^. These results would not only confirm the formation of PSC composite but also affirm the successful surface functionalization of SiO_2_ particles with TMS.

**Figure 2:**
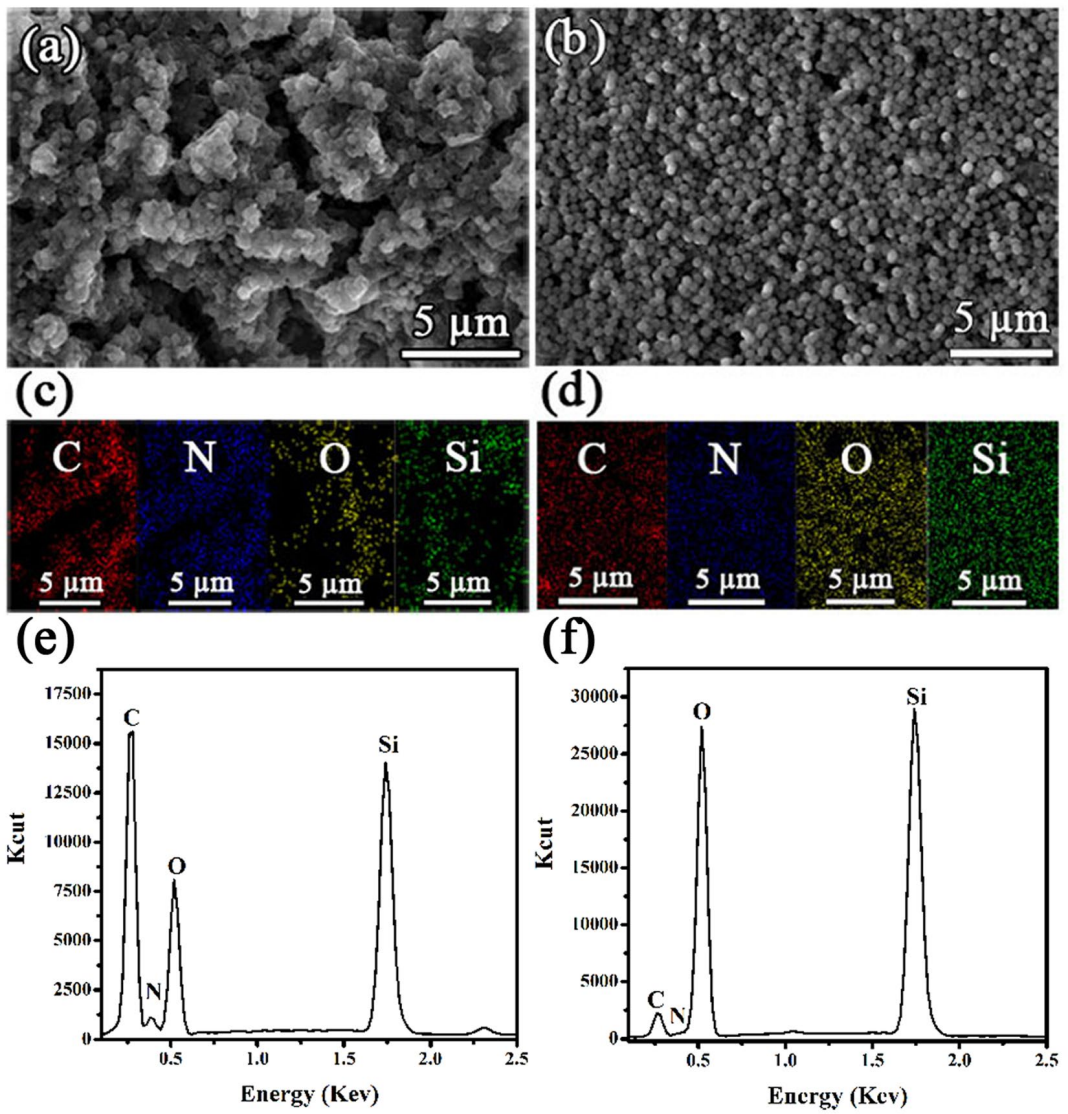
SEM images of (**a**) PDDA/PSC and (**b**) PSC/TMS-SiO_2_ coatings﻿﻿, elemental mappings of (**c**) PDDA/PSC and (**d**) PSC/TMS-SiO_2_ coatings, EDX spectra of (**e**) PDDA/PSC and (**f**) PSC/TMS-SiO_2_ coatings, respectively﻿.

The morphology of silica spheres (SiO_2_) and TMS functionalized SiO_2_ spheres (TMS-SiO_2_) is shown in Fig. S2a and S2b, respectively. The surface of TMS-SiO_2_ particles is still smooth without any aggregation after modification and this transformation is clearly observed in the SEM images with the increase in particle size (Fig. S2b). The SiO_2_ and TMS-SiO_2_ spheres are uniform in size and their size distribution was illustrated in the histograms Fig. S2c and S2d, respectively. The diameter of SiO_2_ and TMS-SiO_2_ is 415 ± 30.28 nm and 495 ± 30.28 nm, respectively, based on 60 randomly selected spheres from Fig. S2a and S2b. It can be seen that in both the histograms the size distribution width is relatively small as compared to previously reported SiO_2_^42, 43^. It is postulated that the morphology of the spheres is in correspondence with the optimized conditions and the equilibrium in particle growth was obtained which results in uniform size of the spheres. Fig. S2e and Fig. 2f shows the SEM images which confirm the formation of PSC composite at low and high magnification, respectively. The synthesized PSC comprises of PANI matrix that exhibits globular morphology with the incorporation of uniformly distributed SiO_2_ (Fig. S2f). These images confirm that the globular space in PANI matrix is occupied by the SiO_2_ particles during chemical oxidative polymerization and these results are in agreement with the previously reported PANI-SiO_2_ composites^43, 44^.

It is important for inorganic nano-particles that they should be homogeneously distributed in the coating matrix; otherwise the fabricated coating would exhibit agglomeration and affect the coating’s long term protection ability. In order to investigate the interaction of SiO_2_ particles with PANI matrix the TEM images were analyzed, as shown in Fig. S3. The SiO_2_ particles were aggregated in ethanol solvent because of the interaction between the hydroxyl groups present on the surface (Fig. S3a and S3b). Contrary to this, the effect of hydroxyl groups associated with SiO_2_ particles in PSC was suppressed with a homogeneous distribution in PANI matrix and the transparent PANI layer connecting the silica particles (dark black spheres) in a network (Fig. S3c). It is clear in the image at high magnification (Fig. S3d), that the PANI layer would make a conductive connection within the SiO_2_ particles and allow them to be homogeneously distributed in a matrix. These properties of the PSC composite in solvent would suggest its potential application as an excellent coating material.

The thermogravimetric curves of PANI, PSC, SiO_2_ and TMS-SiO_2_ were represented in Fig. S4. Generally, the 16% weight loss at 150 °C was due to the presence of moisture in pure PANI. Later on, the deterioration of HCl was noticed within the temperature range of 200–300 °C accompanying with 5% weight loss and the complete degradation of the polymer was observed at 300–650 °C with the final weight loss of 74% (Fig. S4). Contrary to this, SiO_2_ and TMS-SiO_2_ particles show the complete weight loss of 15% and 13%, respectively, which implies that the particles exhibit excellent thermal stability. In case of PSC, 14% weight loss was observed at 150 °C and 3–4% of weight loss between 200–300 °C. The complete degradation of the PSC composite was observed with final weight loss of 35%, which is much less than the weight loss observed for pure PANI. The residue in the temperature range of 500 to 800 °C determines the approximate filler content in PANI matrix^44^ and only 20% weight loss was observed in this region for PSC composite. On the basis of TGA the weight of SiO_2_ particles incorporated in the PANI matrix was estimated to be 45%, which is less than the actual content of the silica particles added in the initial reaction mixture. These results indicate that the addition of SiO_2_ in PANI matrix increases the thermal stability of the PSC and its LbL fabrication with TMS-SiO_2_ particles could be considered as a good candidate for protective coatings.

### Characterization of Coatings

The SEM images of PDDA/PSC and PSC/TMS-SiO_2_ coatings are shown in Fig. 2. It was found that the SiO_2_ particles were homogeneously distributed in PANI matrix, however, the matrix exhibits hierarchical porous network (Fig. 2a). In contrast, the TMS-SiO_2_ layers disguised the hierarchical porosity of PSC and its influence become predominant in the PSC/TMS-SiO_2_ coating (Fig. 2b). The hierarchical porous structure of PDDA/PSC coating influenced the elemental distribution of C, N, O and Si with the inhomogeneous allocation as shown in the EDX mapping (Fig. 2c). However, the homogenous elemental distribution was observed in the PSC/TMS-SiO_2_ coating (Fig. 2d). The elemental EDX spectra shows that the elements associated with PANI i.e., C and N were observed with strong peak intensity in the PDDA/PSC coating (Fig. 2e) as compared to the PSC/TMS-SiO_2_ coating (Fig. 2f). The high intensity peaks associated with oxygen and Si in PSC/TMS-SiO_2_ coating as compared to PDDA/PSC coating implies that the TMS-SiO_2_ layers play a leading role with dominant Si peak intensity. These results indicate that the combination of PSC and TMS-SiO_2_ layers in a multilayer structure would exhibit a synergistic effect of both the layers to produce a uniform protective coating.

**Figure 3:**
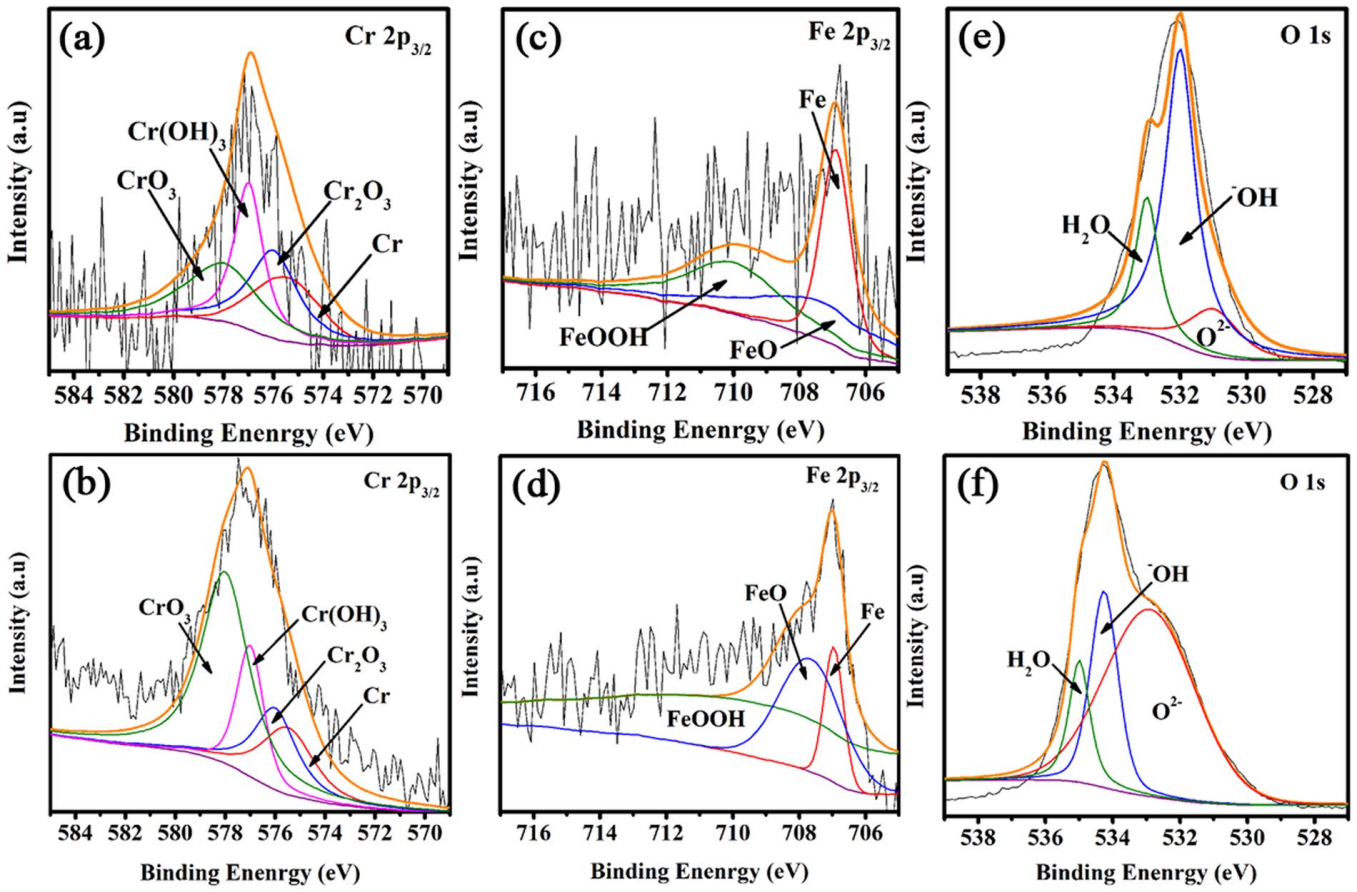
Cr2p3/2, Fe2p3/2, O1s XPS spectra of PDDA/PSC (**a**,**c**,**e)** and PSC/TMS-SiO_2_ coating (**b**,**d**,**f**). To analyze different ionic states, the corresponding elemental fitting of Cr, Fe and O elements were performed as represented by orange lines.

The oxide layer on 316SS surface beneath the PDDA/PSC and PSC/TMS-SiO_2_ coating was analyzed by XPS spectra. The XPS spectra of the oxide layer associated with both the coatings contain Fe2p_3/2_, Cr2p_3/2_, and O1s as the leading constituents^45^, as shown in Fig. 3. The peak at 576 eV in Fig. 3a and b is attributed to the element chromium which was further analyzed to obtain the different ionic states. In case of PDDA/PSC coating, the peak intensity of Cr(OH)_3_ is higher than the Cr_2_O_3_ and same phenomenon was observed in the PSC/TMS-SiO_2_ coating. However, the transformation of Cr(OH)_3_ into Cr_2_O_3_ would take place in PSC/TMS-SiO_2_ coating which results in low intensity of Cr(OH)_3_ and high intensity of Cr_2_O_3_ peak. The presence of pores in PDDA/PSC coating allows water to quickly come in contact with the SS surface which causes a reaction between water and chromium hydroxide^46^. This phenomenon would accelerate the deterioration of the oxide layer in PDDA/PSC coating in contrast to the stable PSC/TMS-SiO_2_ coating.

The peak at about 707 eV is attributed to the element iron and its peak intensity in the oxide layer of PDDA/PSC coating (Fig. 3c) is higher than the PSC/TMS-SiO_2_ coating (Fig. 3d). However, the peak intensity of FeO in PSC/TMS-SiO_2_ coating is high as compared to FeOOH, while an opposite trend was observed in the PDDA/PSC coating. This indicates that the dissolution of iron was occurred in steel alloy during the early stages of anodic passivation leading to the flux of oxides which causes a passivation of residual current^47^.

The peak at about 533 eV in Fig. 3e and f is attributed to oxygen (O1s). The peak intensity of ^−^OH in the oxide layer of PDDA/PSC coating (Fig. 3e) is higher than the PSC/TMS-SiO_2_ coating (Fig. 3f); however, the peak associated with O^2−^ represents the opposite tendency. The peak attributed to water shows high intensity in PDDA/PSC as compared to PSC/TMS-SiO_2_ coating, which implies that the oxide layer beneath the latter one is thicker^48^. The PSC/TMS-SiO_2_ coating is homogeneous therefore thicker oxide layer was formed which hinders the pathway of water and significantly protects the SS surface.

### Contact and Sliding Angle Measurement

**Figure 4:**
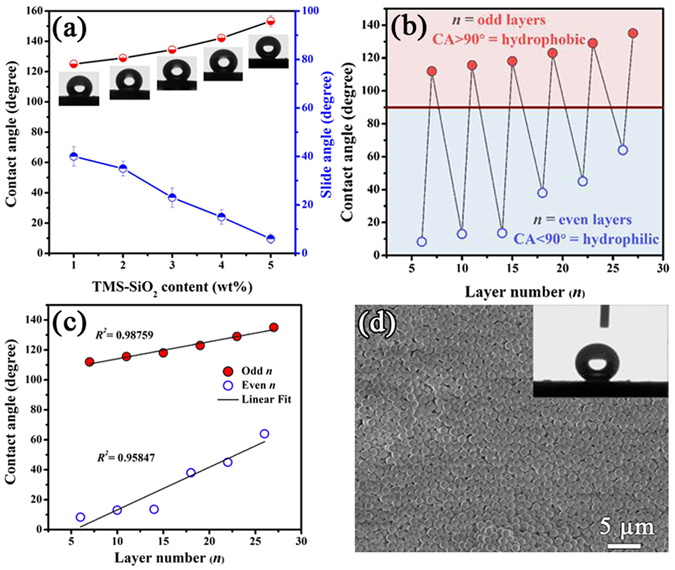
Contact angles (CAs) and sliding angles (SAs) of water droplet on (**a**) PSC/TMS-SiO_2_ coating with different content of TMS-SiO_2_ and corresponding CA images as insets, (**b**) layer number (*n*) modulated reversible surface wettability between hydrophobic and hydrophilic CA as a function of *n*, (**c**) Fitting of CAs at odd and even *n*, (**d**) SEM image of PSC/TMS-SiO_2_ coating with the inset of CA image.

The surface wettability of the coatings was examined by evaluating the contact angles (CAs) of water droplets. The same coating with different regions was dispensed with at least three droplets in order to attain a reliable CAs. Insets of Fig. 4a shows the images of water droplet on PSC/TMS-SiO_2_ coating (at *n* = 29) and increasing trend in CAs was observed with TMS-SiO_2_ content. At 1 wt% TMS-SiO_2_, the coating exhibits hydrophobic behavior with CA = 125° ± 2°, but the increasing content of TMS-SiO_2_ allows the coating to achieve super-hydrophobicity with CA = 153° ± 2° at 5 wt%. The surface wettability of the coating was greatly influenced by TMS-SiO_2_ loading and optimized at 5 wt% because further increase in loading would cause agglomeration and surface defects in the coating.

In addition to CAs, Fig. 4a also represents the sliding angles (SAs) calculated by tilting the PSC/TMS-SiO_2_ surface, sustaining a water droplet until it rolls off. We observed that the surface coated with 5 wt% TMS-SiO_2_ shows extremely low SA, i.e., 6° ± 2° and exhibits the exceptional self-cleaning ability. The SAs decreases with increasing TMS-SiO_2_ content (Fig. 4a), because at low content the particles were irregularly distributed which results in low surface roughness (*S*_a_) of the coating. In contrast, as the content of TMS-SiO_2_ increases the particle distribution on the surface become homogeneous and the roughness reached to tens of micrometers (Table S1). These results suggest that the TMS-SiO_2_ content plays a key role to produce a hierarchical surface with roughness of nano to micrometer scale, thereby results in super-hydrophobic surface with the self-cleaning ability.

### Swapping Behavior of Surface Wettability

Fig. 4b shows the wettability swapping behavior of the PSC/TMS-SiO_2_ multilayers which is tunable with *n*. The surface layer in multilayer coatings determines the CAs, hence the even (6, 10, 14, 18, 22, 26) and odd (7, 11, 15, 19, 23, 27) *n* represent the PSC and TMS-SiO_2_ as a surface layer, respectively. The odd layered surfaces show hydrophobic tendency and the even layered surfaces exhibit hydrophilic tendency. Interestingly, the wettability of the surface is continued to swap between hydrophilic and hydrophobic states with increasing *n*. The LbL multilayer structure of the PSC/TMS-SiO_2_ coating can be tuned in a reversible fashion by controlling *n*. With increasing *n*, it was observed that the hydrophobic character of the coating with odd *n* continues to increase while the hydrophilic character of the coating with even *n* continuously decreases (Fig. 4c). The CA associated with coatings of odd and even *n* was fitted with a straight line having a linear regression coefficient (*R* ^2^) equal to 0.987 and 0.958, respectively. The closely packed TMS-SiO_2_ surface layer can be obtained at *n* = 29 (Fig. 4d) which exhibits super-hydrophobicity and follows the Cassie state^49^ with CA = 153° ± 2° (inset of Fig. 4d).

### Mechanism of Surface Wettability and Swapping

**Figure 5:**
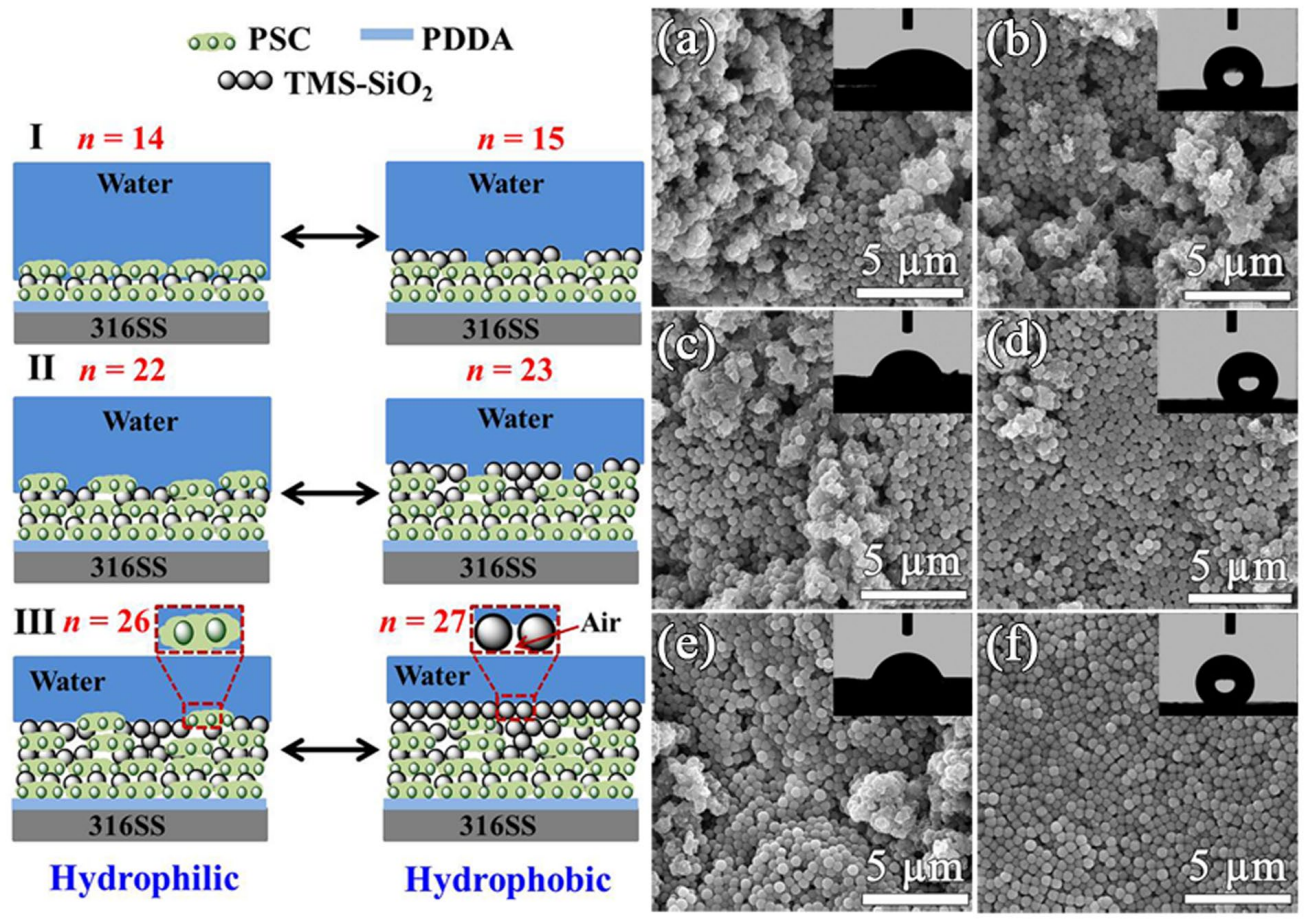
Schematic illustrations of the possible PSC/TMS-SiO_2_ coating-liquid contact modes in regimes I–III with surface wetting reversibility between hydrophilic (*n* = 14, 22, 26) and hydrophobic (*n* = 15, 23, 27) states. SEM image of PSC/TMS-SiO_2_ coating with their CA image as inset at odd *n* (**a**,**c**,**e**) and even *n* (**b**,**d**,**f**).

The CA is a measure of surface wettability of the coating and in case of PSC/TMS-SiO_2_ multilayers it exhibit two different trends^50^, as shown in Fig. 5. The CAs in regime I, II and III at *n* = 14, 22 and 26 displays hydrophilic character (Wenzel state)^51^ due to the presence of PSC as surface layer with pores. However, at *n* = 15, 23 and 27 the TMS-SiO_2_ as surface layer allows the air to trap beneath the water which cause a decrease in solid-liquid contact area and makes the surface hydrophobic (Cassie state)^51^. In regime-I at *n* = 14, the water droplet was quickly spread on the surface with CA = 13.57° ± 1° (inset of Fig. 5a). The porous PSC layer with inhomogeneous hills and valleys (Fig. 5a) increases the solid-liquid contact area and affirms its hydrophilic nature, i.e., fully wetted Wenzel state. This inhomogeneous PSC layer would also affect the deposition of subsequent TMS-SiO_2_ layer (*n* = 15) and its surface wettability. Either the TMS-SiO_2_ surface layer is hydrophobic with CA = 118° ± 2° (inset of Fig. 5b), but due to the presence of underlying porous hydrophilic PSC layer the water would try to diffuse and affects the surface wettability (Fig. 5b). In regime-II at *n* = 22, the PSC surface layer exhibits improved CA = 45° ± 1° (inset of Fig. 5c) as compared to *n* = 14 and this increase was due to the prominent exposure of underneath TMS-SiO_2_ layer (Fig. 5c). At *n* = 23, the surface becomes hydrophobic with CA = 129° ± 3° (inset of Fig. 5d), but the presence of defects preventing the surface to achieve super-hydrophobicity (Fig. 5d). In regime-III at *n* = 26, the increase in CA was observed, i.e., 64° ± 1° (inset of Fig. 5e) due to the dominating effect of underlying TMS-SiO_2_ layer, as seen in Fig. 5e. In contrast, at *n* = 27 the TMS-SiO_2_ layer with increased hydrophobicity CA = 136° ± 2° (inset of Fig. 5f) was due to homogeneous structure and the particles becomes closely packed. This behavior is attributed to the large volume of air held between the water and the TMS-SiO_2_ spheres (Fig. 5, at *n* = 27), hence precludes the intrusion of water droplets within the nanopores^52^.

The average surface roughness (*S*_a_) is one of the most widely used roughness parameter gives the arithmetic average of the absolute values of the roughness profile ordinates^53^. The surface roughness parameter *S*_a_ is available in Detak software which is used to represent roughness and the standard deviation associated with three measured values of the same sample at different positions was presented as error margins in Table S2. The surface morphology of the coating at different *n* reveals that the *S*_a_ and wettability is the inherent property of the material while wettability swap is due to the constituents of the surface layer altered by *n*. The *S*_a_ values of PSC/TMS-SiO_2_ coating were measured with respect to different *n* and summarized in Table S2. The *S*_a_ values are in agreement with the surface morphology represented in Fig. 5a–f; moreover *S*_a_ is a function of *n* and its value decreases with increasing *n* (Table S2). It was observed that the coating at lower *n* i.e., *n = *14 and 15, exhibits high, *S*_a_ values, i.e., 20.17 ± 0.41 and 15.63 ± 0.31 µm, respectively. The difference of roughness between two subsequent layers is high due to the porous and the inhomogeneous structure of PSC layer which is clearly observed in Fig. 5a and b. However, with increasing *n* the surface becomes homogeneous and the effect of TMS-SiO_2_ layer become dominant compared to porous PSC layer. Finally, at *n = *26 and 27, the difference of roughness between two subsequent surface layers becomes small i.e., 11.29 ± 0.34 and 10.03 ± 0.42 µm, because the TMS-SiO_2_ layer become dominant with *S*_a_ values in tens of microns to achieve the super-hydrophobicity (Fig. 5f). The interaction between the PSC layer (hydrophilic) and the underlying TMS-SiO_2_ layer (hydrophobic) lead to different amounts of the layer constituents exposed on the surface at lower *n*. Surprisingly, the wetting phenomenon can be swapped by tuning *n* but the dominant effect of hydrophobic TMS-SiO_2_ layer enhance the hydrophobic behavior consequently decreases the hydrophilic behavior of the coating with increasing *n*. This continuous increase in hydrophobicity would allow the PSC/TMS-SiO_2_ coating to successfully attain a Cassie state with super-hydrophobic surface CA = 153° ± 2° (Fig. 4d). These results imply that the liquid repellency of PSC/TMS-SiO_2_ multilayers can be steadily tuned by varying the loading content of TMS-SiO_2_ and the swapping between hydrophobic and hydrophilic state can be achieved by altering *n*. In addition, the thickness of the coating was also measured with respect to *n* and the values were found to be the function of *n*. The difference of thickness between the two coatings with even and odd *n* is larger at low *n* (i.e., between *n* = 14 and *n* = 15) than the difference at high *n* (Table S2). This is in agreement with the surface roughness data which also shows a high roughness at low *n* and decreases with increasing *n* due to the surface homogeneity achieved by the coating as a function of *n* (Fig. ﻿5a–f).

**Figure 6:**
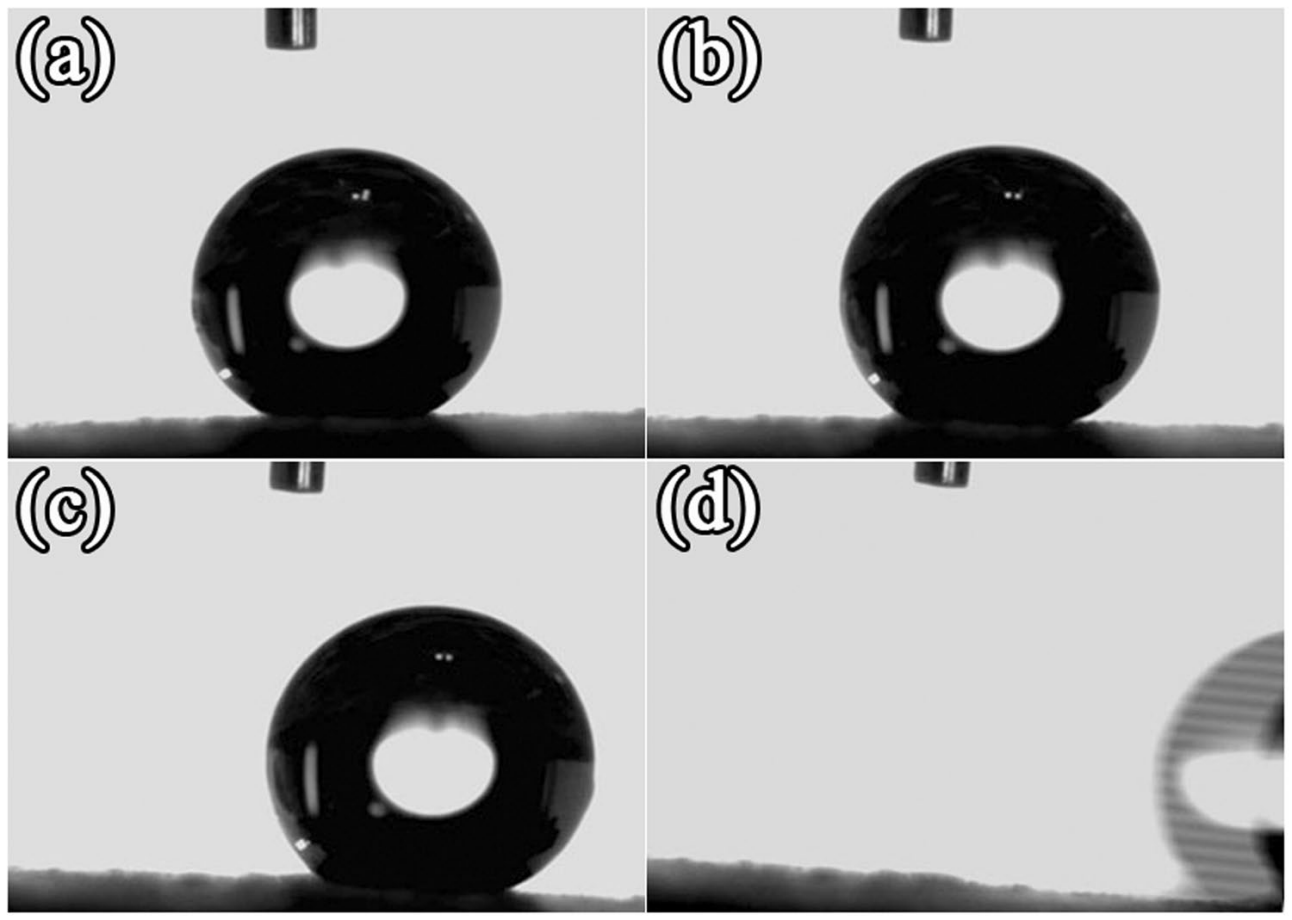
The self-cleaning behavior of water droplet on PSC/TMS-SiO_2_ coating at different SAs (**a**) 0°, (**b**) 2° ± 1°, (**c**) 4° ± 2° and (**d**) 6° ± 2°.

The lotus leaf exhibits the self-cleaning effect which is considered as a unique case of Cassie’s state^51^. This unique property is also termed as “lotus effect” in which the rolling of water droplets would collects the contaminants from the lotus surface and enables its self-cleaning effect^54^. Inspired from this fact, the self-cleaning effect of PSC/TMS-SiO_2_ multilayers was studied. The PSC/TMS-SiO_2_ surface, sustaining a water droplet was tilted at respective SAs and the rolling behavior of the droplet was observed (Fig. 6). Figure 6a shows the PSC/TMS-SiO_2_ surface at SA = 0° and when it was tilted at SA = 2° ± 1° the droplet feels a little force (Fig. 6b) followed by tilting the surface to reach SA = 4° ± 2° (Fig. 6c), here the sliding force becomes saturated. The maximum SA required by the PSC/TMS-SiO_2_ surface to roll off the water droplet is 6° ± 2°, as shown in Fig. 6d. The PSC/TMS-SiO_2_ coating with CA = 153° ± 2° (supported by the inset of Fig. 4d) exhibits super-hydrophobicity and its water repellent rough surface with low SA of 6° ± 2° showcase the coating’s self-cleaning ability. These results imply that, the solid-liquid contact area was minimized that allows the formation of a spherical water droplet to rolls off at SA = 6° ± 2° (Fig. 6d) taking the contaminant particles with it. In addition, Video S1 also demonstrates the sliding behavior of PSC/TMS-SiO_2_ surface and the water droplet will eventually roll off at SA = 6° ± 2°. If the coating surface is stabilized at the sliding angle of 8°, there is a continuous rolling of water droplet was observed as shown in Video S2. To further support the rolling behavior of water droplet, we captured a real time Video S3. In this video, the PSC/TMS-SiO_2_ coated 316SS coupon was placed on the tilted glass slide with approximate SA of 10° ± 3° and water was continuously dropped with a micropipette. It was observed that the water droplet continues to slide on the surface without resistance owing to the super-hydrophobicity and self-cleaning ability of the coating. These results reflect that the excellent self-cleaning ability of the coating could be achieved by reducing the adhesion of water droplets at the solid-liquid interface.

### Physical properties of the coating

The primary feature to satisfy the advanced anti-corrosion performance of the coating is the superior physico-mechanical properties^55, 56^. The adhesion and barrier properties of the coatings are presented in Table 1. The films with 1 wt% and 2 wt% TMS-SiO_2_ show high degree of adhesion due to the dominant effect of PSC as observed in the similar cases previously reported^55^, leading to improved compatibility with the substrate. However, with increasing content of TMS-SiO_2_ i.e., 3–5 wt% a slight decrease in adhesion degree was observed as compared to 1–2 wt%. The combination of TMS-SiO_2_ with PSC reduces the elasticity and ductility effect attributed to coating matrix, hence maintains the mechanical strength of the films. These results imply that the addition of TMS-SiO_2_ makes the coating harder with good mechanical strength due to virtuous compatibility with the composite.

**Table 1:** The physical properties of PSC/TMS-SiO_2_ film as a function of TMS-SiO_2_ content.

TMS-SiO_2_ content (wt%)	Adhesion degree	Oxygen permeability (%)	Vapor permeability (g/hm^2^)
1	4B	0.75	96.12
2	4B	0.64	84.57
3	3B	0.55	77.39
4	3B	0.42	64.24
5	3B	0.34	55.19

The optimum size and shape of the particles along with their homogenous distribution in the coating matrix affect the barrier properties of the coating^57^. The gas and water vapor permeability of PSC/TMS-SiO_2_ free standing films are summarized in Table 1. It was found that in the presence of 1 wt% TMS-SiO_2_ the film exhibits high oxygen and water permeability i.e., 0.75% and 96.2 g/hm^2^, as compared to 5 wt% TMS-SiO_2_ film which shows a remarkable reduction in molecular permeability values 0.34% and 55.19 g/hm^2^. The molecular permeability of the films is a function of TMS-SiO_2_ content and its presence in the coating increases the tortuosity of diffusion pathway of oxygen and water molecules^58^. The profound increase in barrier ability was attributed to good compatibility of the composite and TMS-SiO_2_ spheres dispersed as nanolayers in the composite^57, 58^.

To determine the influence of pH on surface wettability and durability of the PSC/TMS-SiO_2_ coating, the samples (at *n* = 29) were immersed in a solution of pH range 1–5 (pH adjusted with 0.1 M HCl) for 15 min followed by vacuum drying. The change in surface wettability parameters of pretreated samples as a function of pH are presented in Table S3, each reading is an average of three values. The hydrophobic character of the coating increases with pH, at pH = 1 the coating exhibits hydrophobicity with CA = 110° ± 3.3° and SA = 55° ± 1.65°, as the pH of the solution become less acidic (pH = 5), the surface recovers its super-hydrophobicity with CA = 151° ± 1.5° and SA = 9° ± 0.45°. This change in hydrophobic character is due to decrease in surface roughness and transformation in surface hydration process under the influence of acidic pH. However, the coating maintains its hydrophobicity even after being exposed to acidic medium which indicates the durability of the coating.

### Electrochemical and Corrosion Resistance Measurements

The electrical activity of the coated and uncoated samples was recorded as cyclic voltammograms. As shown in Fig. S5a, the absence of any peak in the potential range 0–1 V of uncoated samples indicate lack of redox activity. In contrast, the voltammogram of the PSC/TMS-SiO_2_ coating exhibits oxidation peak, which is attributed to the transformation of emeraldine salt (ES) to pernigraniline base (PB) in the potential range 0.65–0.80 V. The mechanism involved in this transformation is shown in Fig. S5b and the reduction peak during the cathodic scan is attributed to the reversal process (0.2–0.4 V). Although, there is a combination of PSC layer with subsequent TMS-SiO_2_ layer in a multilayer structure, but the coating still maintains the electroactivity of PANI polymer. Because of its redox catalytic properties, conjugated PANI considered as a potential material for anti-corrosion coating that allows the formation of a passive metal oxide layer and offers an effective corrosion resistance for metals^59^. We therefore envisioned that the PSC/TMS-SiO_2_ coating significantly enhanced the corrosion resistance due to the synergistic effect of passive metal oxide layer and super-hydrophobicity which offers an effective barrier against the invasion of aggressive media (as supported by physical properties).

**Figure 7:**
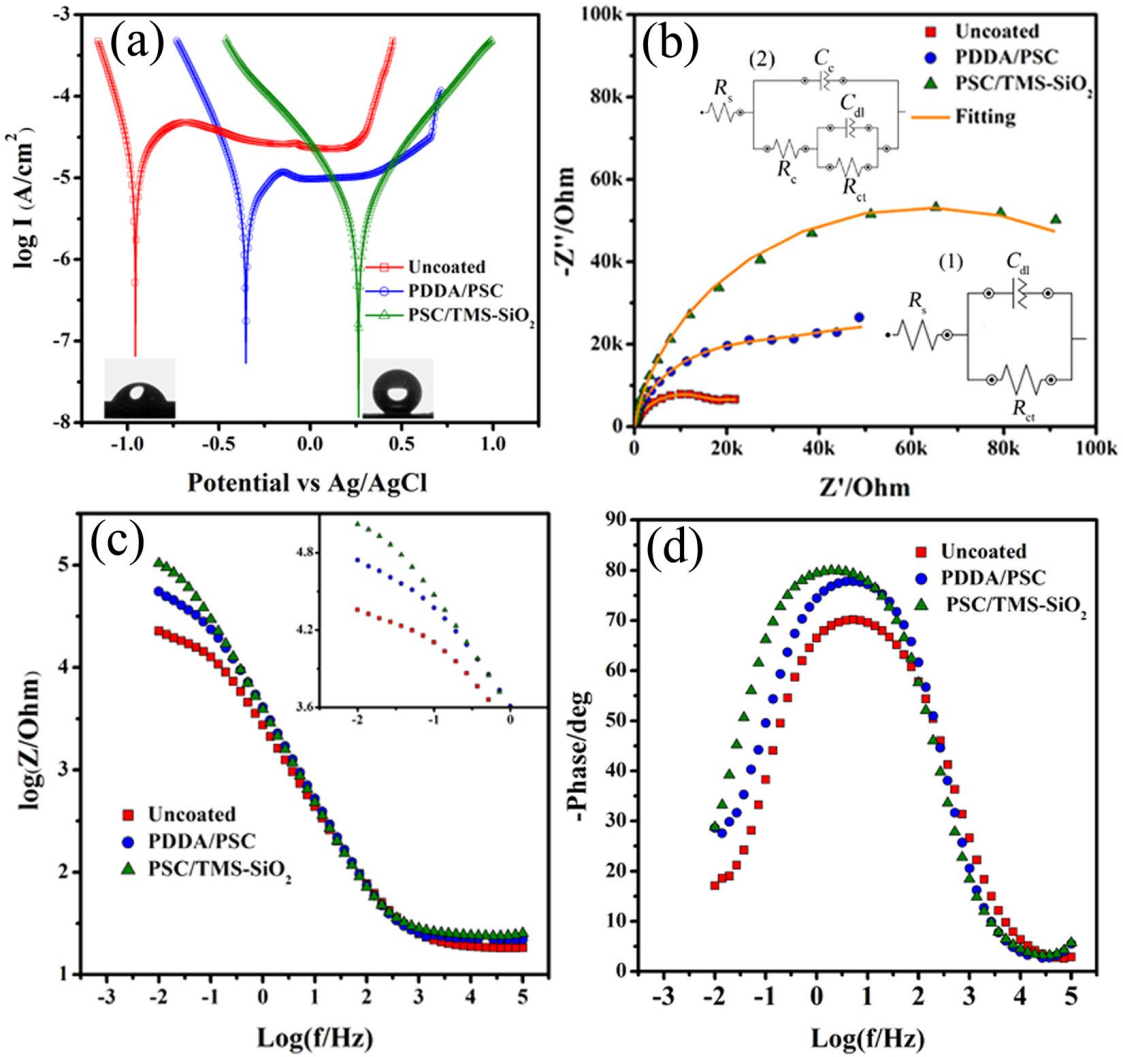
Potentiodynamic polarization curves of (**a**) uncoated, PDDA/PSC and PSC/TMS-SiO_2_ coating with their respective CA images as inset. (**b**) Nyquist plot of uncoated and coated samples, equivalent circuits used to fit the EIS diagrams of (b1) uncoated and (b2) coated samples. (**c**) Bode impedance and (**d**) phase plot of uncoated and coated samples.

The polarization curves of uncoated and coated 316SS in 3.5% NaCl is shown in Fig. 7a with their respective CA images as insets. The PDDA/PAA coating is super-hydrophilic therefore, its CA image is not mentioned. The corrosion parameters such as corrosion potential (*E*_corr_) and corrosion current (*I*_corr_) obtained through these curves are summarized in Table 2. For a typical polarization curve, the high *E*_corr_ and a low *I*_corr_ is attributed to a better corrosion resistance and a low corrosion rate. From these polarization curves, it can be seen that the *E*_corr_ = −352 of PDDA/PSC coating is more positive as compared to the uncoated 316SS with *E*_corr_ = −957. The transformation between different states of PANI i.e., emeraldine salt (ES) to leucoemeraldine (LE) causes a positive shift in *E*_corr_ values because the nonconductive LE retards the electron transport between SS and coating^60, 61^. The potential range of uncoated samples is −0.65 to 0.27 V which indicates the presence of a passive oxide layer, but this passivation crumpled at 0.27 V with a sudden increase in current (Fig. 7a). In case of PDAA/PSC coating the passivation plateau range becomes more positive, i.e., −0.15 to 0.65 with a crumpled passivation at 0.65 V. In contrast, there is no passivation plateau and no sharp increase in corrosion current was observed in case of super-hydrophobic PSC/TMS-SiO_2_ coating which implies that the coating maintains the barrier against the corrosive species and exhibits enhanced corrosion protection ability. The *I*_corr_ was reduced from 40.06 (uncoated SS) to 7.31 µA cm^−2^ for PDDA/PSC coating. Overall, the polished 316SS with CA 64.7° ± 1° (inset of Fig. 7a, uncoated SS) presents low *E*_corr_ and a high *I*_corr_ value which indicates that the surface is more prone to corrosion. However, the super-hydrophobic PSC/TMS-SiO_2_ coating achieved highest *E*_corr_ = −263 and the lowest *I*_corr_ = 2.09 µA cm^−2^ which shows the superior anti-corrosion ability of the coating as compared to its hydrophilic and uncoated counterparts. The protective efficiency (*P*_*e*_) of the coating can be expressed by the following equation:

1 $${P}_{e}=100\times (1-\frac{{i}_{corr}}{{i}_{corr}^{^\circ }})$$

where *i*_corr_ and *i* ^*°*^_corr_ are the current densities in the presence and absence of the coating, respectively. The comparison between PDAA/PSC and PSC/TMS-SiO_2_ coating shows that the latter exhibits *P*_*e*_ = 94.78% (Table 2), which is greater than the former hydrophilic coating with *P*_*e*_ = 81.75%. The main reason behind the enhancement in corrosion resistance is the “lotus effect” and this effect is produced due to the textured super-hydrophobic surface with nano-porous structure (supported by Fig. 5), which impedes the penetration of water and aggressive species to approach the SS surface.

**Table 2:** The corrosion parameters obtained from polarization curves of uncoated and coated 316SS.

Sample	*E*_corr_ (mV)	*I*_corr_ (μA cm^−2^)	*P*_*e*_ (%)	CA (°)
Uncoated	−957	40.06	—	64.7° ± 1°
PDDA/PSC	−352	7.31	81.75	—
PSC/TMS-SiO_2_	−263	2.09	94.78	153° ± 2°

Electrochemical impedance spectroscopy (EIS) was used as an alternative method to evaluate the corrosion protection ability of the coatings. Fig. 7b shows the Nyquist plot and Fig. 7c,d﻿ shows the Bode plot of the PDDA/PSC, PSC/TMS-SiO_2_ coating and uncoated 316SS in 3.5% NaCl solution. The Nyquist plot of the coated samples, exhibits a high impedance with single capacitive loop than the uncoated ones. The corrosion resistance of different samples can be quantified by fitting the EIS data using equivalent circuits which provide the selective information about the permeability of the corrosive species involved in coating degradation^62^. The equivalent circuits used for uncoated and coated samples are shown in Fig. 7b1 and b2. These equivalent circuits consist of charge transfer resistance (*R*_ct_) and solution resistance (*R*_s_) with double layer capacitance (*C*_dl_)^63^. For coated samples, the equivalent circuit added with the coating capacitance *C*_c_ and resistance *R*_c_. The double layer behavior was expressed as constant phase element (*CPE*) in the electrical equivalent circuit and used when a deviation was observed from the pure-capacitive behavior to obtain a better simulation and it is expressed in the following equation as^64^:

2 $${Z}_{CPE}=\frac{{\rm{1}}}{{\rm{Q}}{({\rm{j}}\omega )}^{{\rm{\alpha }}}}$$

where *CPE* is the *Q*, *j* and *ω* is the imaginary unit (square = −1) and the angular frequency (*ω* = *2πf*) respectively, the ideal capacitance is unity but the deviation is expressed as (0 < α < 1). The parameters obtained through circuit fitting are summarized in Table 3 and a good fitting was achieved by using the suggested equivalent circuits. The values of the exponent (n) are within the range of 0.6–0.7 which indicates that *CPE*_c_ and *CPE*_dl_ can be expressed as capacitance *C*_c_ and *C*_dl_^65^.

**Table 3:** Electrochemical parameters of uncoated and coated 316SS in 3.5% NaCl solution.

Sample	*R*_s_ (Ωcm^2^)	*C*_c_ (F/cm^2^)	*R*_c_ (kΩcm^2^)	*C*_dl_ (F/cm^2^)	*R*_ct_ (kΩcm^2^)	*R*_p_ (kΩcm^2^)
Uncoated	18.39	—	—	8.09 × 10^−5^	22.97	22.97
PDDA/PSC	22.43	4.53 × 10^−5^	14.96	1.71 × 10^−5^	43.00	57.96
PSC/TMS-SiO_2_	25.06	4.41 × 10^−5^	27.25	1.37 × 10^−5^	99.23	126.48

Generally, the barrier ability of the coating is expressed by the coating resistance (*R*_c_) and capacitance (*C*_c_)^66^. In Table 3, the PDDA/PSC coating exhibits low *R*_c_ and high *C*_c_ values which involve the quick diffusion of electrolyte solution due to hydrophilic nature of the coating. In contrast, for PSC/TMS-SiO_2_ coating the *R*_c_ values increased from 14.96 (PDDA/PSC coating) to 27.25 kΩcm^2^ with low *C*_c_ values. The high *R*_c_ with low *C*_c_ value indicates the superior barrier ability due to super-hydrophobicity which blocks the permeation of corrosive media and shows the excellent corrosion protection ability. The element *R*_ct_ in the equivalent circuit represents the electron transfer across a metal surface. For uncoated samples *R*_ct_ signifies the polarization resistance (*R*_p_) and for coated samples it is the combination of *R*_c_ and *R*_ct_^67^. Therefore, we used *R*_p_ to compare the corrosion protection ability of the coatings and it was found that the *R*_p_ value of PDDA/PSC coating is 57.96 kΩcm^2^ which is two times higher than the uncoated sample i.e., 22.97 kΩcm^2^. This increase is attributed to the physical barrier provided by the coating due to the presence of SiO_2_ spheres and insoluble species formed during redox reactions of PANI. In case of super-hydrophobic PSC/TMS-SiO_2_ coating high *R*_p_ value (126.48 kΩcm^2^) indicates that the TMS-SiO_2_ increases the tortuosity in diffusion pathway of corrosive species thus enhances the corrosion resistance of the coating^68^.

The Bode impedance (Fig. 7c) and phase (Fig. 7d) plot also supports the fact that the coated SS exhibits good anti-corrosion ability as compared to the uncoated ones. The protective properties of the coating is represented by the impedance modulus at low frequencies^69^ as shown in Fig. 7c. In this region, the PSC/TMS-SiO_2_ coating shows significantly high impedance value of the order >100 kΩcm^2^. The inset of Fig. 7c shows that the absolute impedance of the PSC/TMS-SiO_2_ coating is higher than the PDDA/PSC coating and uncoated samples. In case of ideal capacitance the phase angle (α) and slope (S) is −90° and −1, respectively^70^. The α associated with the linear regions in the Bode impedance diagram of PDDA/PSC and PSC/TMS-SiO_2_ coating is −78° and −82° with a slope of −0.84 and −0.87 (Fig. 7d). Among these two, the PSC/TMS-SiO_2_ coating is closer to the ideal values of α and S; hence contribute to the superior anti-corrosion performance.

The integrity and aesthetic appearance of the PSC/TMS-SiO_2_ coating was studied by making a cross-cut on the surface with scalpel and exposed to 3.5% NaCl solution. Fig. S6a and S6b show the optical microscopic image of the scribed coating before and after immersion in 3.5% NaCl for 100 h. After immersion, the coating maintains its overall surface integrity, but in the vicinity of the cross-cut delamination (the red circled area Fig. S6b) occurred due to the continuous diffusion of electrolyte during 100 h of immersion. Fig. S6c shows the Nyquist plot of the scribed coating elaborates the 24 hourly monitored electrochemical changes during 100 h of immersion in chloride containing electrolyte. Generally, the *R*_ct_ of the coating decreases abruptly if a crack appears on the coating, due to the quick diffusion of the electrolyte leading to direct contact with the bare SS surface. The *R*_ct_ of the scribed PSC/TMS-SiO_2_ coating at 0 h of immersion is 140.45 kΩcm^2^. Although, during 100 h of immersion the *R*_ct_ value continuously decreases, but the coating still maintains its *R*_ct_ value, i.e., 105.12 kΩcm^2^ even after 96 h of immersion with no abrupt decrease. These *R*_ct_ values of the scribed coating are roughly similar to the coating in the absence of the scribe (compared to *R*_ct_ values in Table 4). The reason behind this is the coating could maintain its integrity due to the TMS-SiO_2_ layer with super-hydrophobic characteristics which provides a strong barrier against further electrolyte diffusion in the vicinity of the cross-cut and PANI would form an oxide layer (supported by XPS data). These results imply that the coating could maintain its integrity, aesthetic appearance, adhesion and protect the underlying SS surface in chloride containing environment even in the presence of a scribe.

**Table 4:** Electrochemical parameters obtained by fitting the EIS data of uncoated SS and PSC/TMS-SiO_2_ coating with their respective contact angles (CAs) at different immersion time in 3.5% NaCl.

Time (h)	Uncoated 316SS			PSC/TMS-SiO_2_ coating						
	*R*_s_ (Ωcm^2^)	*C*_dl_ (F/cm^2^)	*R*_ct_/*R*_p_ (kΩcm^2^)	*R*_s_ (Ωcm^2^)	*C*_c_ (F/cm^2^)	*R*_c_ (kΩcm^2^)	*C*_dl_ (F/cm^2^)	*R*_ct_ (kΩcm^2^)	*R*_p_ (kΩcm^2^)	CA (°)
1	23.71	1.78 × 10^−4^	30.95	60.62	4.21 × 10^−5^	78.49	1.06 × 10^−4^	139.37	217.86	152° ± 2.3°
24	22.88	1.79 × 10^−4^	27.46	66.68	9.07 × 10^−5^	68.87	1.17 × 10^−4^	129.32	198.19	149° ± 1.5°
48	23.47	1.81 × 10^−4^	27.21	65.18	9.37 × 10^−5^	67.24	1.39 × 10^−4^	117.23	184.47	146° ± 2.1°
72	20.29	1.83 × 10^−4^	26.40	65.38	9.82 × 10^−5^	66.30	1.59 × 10^−4^	116.19	182.49	142° ± 2.5°
96	21.35	1.85 × 10^−4^	23.94	60.17	9.89 × 10^−5^	64.85	1.61 × 10^−4^	115.39	180.24	137° ± 3.2°
120	20.84	1.89 × 10^−4^	23.11	63.81	9.92 × 10^−5^	63.98	2.41 × 10^−4^	111.60	175.58	129° ± 1.4°
144	21.11	1.91 × 10^−4^	22.58	64.44	9.94 × 10^−5^	63.74	2.50 × 10^−4^	99.51	163.25	125° ± 2.8°
168	21.75	1.93 × 10^−4^	19.96	60.03	1.08 × 10^−4^	62.45	2.81 × 10^−4^	98.72	161.17	115° ± 2.6°
192	20.73	1.99 × 10^−4^	19.82	62.22	1.10 × 10^−4^	61.10	2.90 × 10^−4^	92.59	153.69	105° ± 3.3°
216	21.76	2.14 × 10^−4^	17.41	64.36	1.20 × 10^−4^	59.23	6.36 × 10^−4^	86.21	145.44	101° ± 2.2°
240	21.22	7.71 × 10^−4^	17.61	63.58	1.29 × 10^−4^	55.75	9.81 × 10^−4^	77.81	133.56	98° ± 1.9°

### Effect of Long Term Exposure

**Figure 8:**
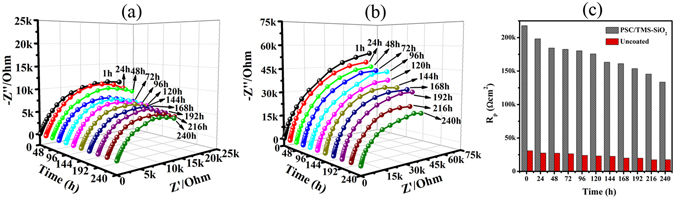
Nyquist plots of (**a**) uncoated 316SS and (**b**) PSC/TMS-SiO_2_ coating exposed to 3.5% NaCl for 240 h, (**c**) change in *R*_p_ during 240 h of exposure of uncoated and coated samples with thier comparison.

The anti-corrosion behavior of the coating was illustrated in the previous section on the basis of EIS data (Fig. S6c and Fig. 7), but this information is not quite enough to illustrate the lifespan and durability of the coating. In order to understand in detail the degradation mechanism and long term anti-corrosion ability of the coating, we performed EIS for extended immersion time. Figure 8a and 8﻿b represents the Nyquist plot of the uncoated SS and PSC/TMS-SiO_2_ coating in 3.5% NaCl for 240 h. Both the samples exhibit single capacitive loop while the PSC/TMS-SiO_2_ coating shows enhanced corrosion resistance and this property is stable even after 240 h of immersion in aggressive environment.

The obtained EIS parameters are summarized in Table 4. During 1 h of immersion the lowest *C*_c_ value was observed due to the super-hydrophobic property of the coating which does not allow the water and corrosive species to permeate. However, as the immersion time increases the corrosive species would approach the micro and nano-pores to diffuse into the coating this phenomenon is supported by the gradual increase in *C*_c_ values. The change in *C*_c_ values is very small within 24–96 h of immersion and there is no drastic increase in the values due to the subsequent TMS-SiO_2_ layers which hinders the diffusion of aggressive species. At exposure time 120–192 h, there is a gradual increase in *C*_c_ values due to the dominant hydrophilic effect of PSC layers. Finally, at 216–240 h of immersion an accelerated diffusion of corrosive species was observed supported by the increase in *C*_c_ values. In spite of this, the oxide layer on the SS surface in the presence of PANI matrix tries to maintain the anti-corrosion ability of the coating. The *R*_ct_ values of PSC/TMS-SiO_2_ coating gradually decrease with the immersion time, but the coating maintains the passivity of the SS surface as compared to uncoated samples.

To further explain the protection mechanism of the coating, polarization resistance (*R*_p_) was used and Fig. 8c shows the contrast between the *R*_p_ of the uncoated SS and PSC/TMS-SiO_2_ coating. For uncoated SS, the *R*_p_ values gradually decrease from 30.95 to 17.61 kΩcm^2^ and the direct contact of aerated solution with SS surface results in the formation of an oxide layer. This layer would slowly damage during 240 h of exposure in a 3.5% NaCl solution (Table 4). In contrast, the *R*_p_ value of the PSC/TMS-SiO_2_ coating remains high after 1 h of immersion due to water repelling ability of the coating. After 24 h the slight decrease in the *R*_p_ value was associated with the permeation of aggressive chloride ions that affect the water repelling property of the coating. This phenomenon was attributed to the slow loss of the surface hydrophobicity and uptake of aerated corrosive solution. In spite of this, the *R*_p_ values of the PSC/TMS-SiO_2_ coating were higher than the uncoated samples throughout the exposure time. At 48–96 h of immersion, the *R*_p_ values stabilized within the range of 117.23–115.39 kΩcm^2^ followed by a gradual decrease in *R*_p_. The stability is associated with the synergistic barrier ability of electro-active PSC and subsequent TMS-SiO_2_ layers. Further increase in exposure time, i.e., 144–240 h results a little decease in *R*_p_ values, however, the transformation between different PANI oxidation states and its redox catalytic behavior was activated. As a result, PANI would attain a positive potential in emeraldine state which passivates the SS surface and hence stabilized the corrosion process^71^. In addition, the diffused oxygen would reached the coating/metal interface which result in the formation of metal oxide layer (supported by XPS data) and maintains the anti-corrosion ability during prolonged exposure^59^. However, the high *R*_p_ values and a gradual decrease with immersion time suggest that the coating prevents the sudden breakdown of SS surface and maintains the corrosion resistance for longer time. Moreover, the change in CAs of PSC/TMS-SiO_2_ coating surface with immersion time in 3.5% NaCl solution was measured and summarized in (Table 4). Initially, the coating maintains the super-hydrophobicity even being exposed to chloride containing environment, but after 96 h of immersion the electrolyte trying to diffuse into the coating consequently affect the surface wettability of the coating. Therefore, the CA values decreases with increasing immersion time and reached to 98° ± 1.9° at 240 h of immersion. Importantly, the coating still maintains the hydrophobicity and stabilized the quick degradation of the coating during prolonged immersion and protects the underlying metal.

The effect of long term immersion on morphology of coated and uncoated PSC/TMS-SiO_2_ coating was further studied through visual screening. Fig. S7a and S7b show the digital images of the polished and coated 316SS before immersion in 3.5% NaCl. The Fig. S7c and S7d shows the red dotted line encircled area of diameter 1 cm is exposed to 3.5% NaCl for 240 h. It can be seen that severe pitting corrosion was initiated with the appearance of dots which can clearly be seen in the magnified microscopic image of polished SS surface (Fig. S7e). In contrast, the PSC/TMS-SiO_2_ coating sacrifices itself during prolonged immersion time in saturated chloride media and cracks were originated, but there is no delamination and quick decrease in resistance was observed even after 240 h of immersion (supported by Fig. 8b). These microscopic images reveal that the coating significantly protects the underlying SS surface, even after 240 h of immersion by sacrificing itself and increases the lifespan of SS in corrosive media.

From these observations we conclude that different mechanism involved in the prolonged corrosion protection ability of the PSC/TMS-SiO_2_ coating: (i) the super-hydrophobicity of the coating enhances the water repellent property, (ii) the good barrier ability of the coating was attributed to the combination of PSC and TMS-SiO_2_ in a multilayer structure, (iii) PANI maintain its emraldine state with anodic passivation during the redox transformation and its presence at the coating/metal interface would result in the formation of oxide layer^64^. It is worthwhile to point out that the PSC/TMS-SiO_2_ coating maintains its aesthetic appearance during prolonged exposure of 240 h and enhances the corrosion resistance of the SS (supported by electrochemical studies). These super-hydrophobic coatings are potential candidates for prolonged corrosion protection of metal and can be used where tunable surface wettability and swapping between the hydrophobicity and hydrophilicity is desired.

## Conclusions

In this work, a facile approach was used to impart super-hydrophobicity on the SS surface by integrating two materials with different surface energy, i.e., PSC and TMS-SiO_2_ in LbL multilayer fashion by using spin assembly. The unique integration of two materials leads the coating’s surface to swap between hydrophobic and hydrophilic state by tuning *n* and this swapping can be controlled over a wide range. The high CA (153° ± 2°) with very small SA (6° ± 2°) reveals the super-hydrophobic and self-cleaning ability which is due to the synergy of surface composition and roughness of the coating. The super-hydrophobic PSC/TMS-SiO_2_ multilayer coating exhibits enhanced corrosion resistance compared to hydrophilic PDDA/PSC coating. Interestingly, the long-term EIS results reveal that the high corrosion resistance of the coating is stable even after 240 h of immersion in 3.5% NaCl and no drastic degradation was observed as compared to uncoated samples. The excellent corrosion protection was attributed to the synergistic effect of super-hydrophobicity and redox catalytic behavior of PANI. Different mechanisms were proposed for long-term protection, including excellent barrier ability and water repellent property of TMS-SiO_2_ layer in combination with PANI that induce an anodic passivation followed by the formation of an oxide layer. This type of coating is a potential candidate for long-term protection in corrosive environment and it can be employed as a conventional engineering material where swapping in surface wettability is required.

## Methods and Experimental Section

### Materials

Tetraethylorthosilicate (TEOS), absolute ethanol, Hexamethyldisilazane (HDMS) and ammonia (25%) were received from Sinopharm Chemical Reagent. Aniline monomer (ANI), ammonium persulphate (APS), N-methyl-2-pyrrolidone (NMP) and hydrochloric acid (HCl) were purchased from Shanghai Lingfeng Chemicals. Polyacrylic acid (PAA) of average Mw ~ 450,000 and polydiallyldimethylammonium chloride (PDDA) of average Mw ∼ 200,000–350,000 were purchased from Sigma-Aldrich. The 316SS coupons (2.5 × 2.5 × 0.1 cm) were supplied by Senda decoration materials (China). The coupons were polished with different grades of SiC papers up to 1200 and then the surface was polished with 3μm alumina slurry. Prior to coating, the coupons were washed in deionized water and dried.

### Synthesis of Silica NPs

The uniform sized mono dispersive silica particles (SiO_2_) were synthesized via well-known Stober method^72^ and the molar proportion of TEOS, water, ethanol and ammonia was 1:14:28:0.5. Initially, deionized water, absolute ethanol and ammonia were stirred in a beaker for 10–15 minutes to obtain a homogeneous solution. The hydrolysis of TEOS (1 M) were carried out by adding it in the as-prepared solution mixture and stirred for the next 24 h to obtain a milky suspension. The SiO_2_ particles were separated by centrifugation and washed with deionized water 3–4 times followed by vacuum drying at 70 °C.

### Synthesis of PANI-SiO_2_ Composite (PSC)

The PANI and SiO_2_ composite were prepared by oxidative polymerization of aniline in the presence of SiO_2_. 1 ml of HCl solution was prepared in a conical flask and SiO_2_ particles were added with sonication for 10–15 minutes, followed by a vigorous stirring for 1 h to homogeneously disperse silica particles. Then the temperature of the solution mixture was maintained at 0–5 °C using an ice bath with the addition of (0.014 M) ammonium persulphate (APS). Finally, aniline monomer was added drop-wise within 30 minutes to the reaction mixture and the weight ratio of aniline to SiO_2_ was 1:1. The reaction mixture was left for 6 h with vigorous stirring to get complete polymerization of aniline monomer and its composite formation with SiO_2_. After polymerization, the synthesized PSC composite was isolated by centrifugation and washed three times with deionized water followed by overnight vacuum drying at 60 °C.

For the preparation of coating solution, 0.05 mM of PAA was added in 100 ml of NMP the solution was vigorously stirred until the solution become homogeneous. Then 5% wt/v of PSC was added in the solution and stirred for 24 h to get a uniformly dispersed composite prior to multilayer fabrication. The preparation scheme of PSC composite and its coating solution is shown in Fig. 1a.

### Modification of SiO_2_ particles

The surface of as synthesized SiO_2_ particles were functionalized with trimethylsiloxane (TMS) according to as reported procedure^40, 73^. 3 g of silica particles was dispersed in 50 ml of methyl isobutyl ketone followed by a stirring of 30 minutes at 50 °C. Then the solution was placed at 50° with the addition of 100 ml HDMS and stirred continuously for 24 h. The TMS functionalized silica particles (TMS-SiO_2_) were dispersed in toluene solution (5% wt/v) to obtain a coating solution which is used for the fabrication of multilayers. The surface functionalization of SiO_2_ and preparation of its coating solution is presented in Fig. 1b.

### Fabrication of LbL PDDA/PSC and PSC/TMS-SiO_2_ Multilayers

LbL multilayer films were fabricated on 316SS surface by spin-coating, as reported previously^35^. Two types of coating i.e., PDDA/PSC and PSC/TMS-SiO_2_ were prepared to compare the surface wettability and anti-corrosion performance. As shown in scheme (Fig. 1c), 1 ml of PDDA solution (2 mg/ml) casts on the substrate. The substrate was rotated at slow speed of 500 rpm for 30 sec in order to cover the surface and the edges of the substrate with PDDA solution. The substrate was then accelerated at a speed of 1000 rpm for 1 minute and the excess of liquid flies off the surface due to centrifugal force. Secondly, PSC layer was fabricated by following the same deposition procedure used for PDDA layer. After deposition of each layer the substrate was heated on the temperature controlled hot plate at 110 °C for 30 sec. This heating step would evaporate the solvent trapped during the deposition step and results in the formation of a uniform coating. Repetition of the subsequent deposition steps result in the formation of multilayers with different *n* (Fig. 1c). For the preparation of LbL PSC/TMS-SiO_2_ multilayers, the same fabrication procedure was followed (Fig. 1d), except the first five layers of PDDA, which were deposited in order to achieve better adhesion to the substrate.

### Instrumentation

The multilayer structure was obtained by KW-4A/5 spin coater and Fourier transform infrared (FTIR) spectra were recorded on the Spectrum-GX of Perkin Elmer. The morphologies and structure of the synthesized material and the coating were analyzed via scanning electron microscope (SEM) of Hitachi S-4800 and JEOL JEM-2100 transmission electron microscope (TEM). Energy dispersed X-ray spectroscopy (EDX) was performed by the same SEM and for accuracy the atomic percentages were averaged from repeated EDX measurements at different positions. NETZSCH STA-409-PC instrument was used to perform thermogravimetric analysis (TGA). The coating was carefully removed by immersing the coated samples in 3.5% NaCl solution for 24 h and the underlying surface of 316SS was monitored by X-ray photoelectron spectroscopy (XPS) of Thermo Fisher Scientific K-Alpha to analyze the underlying metal oxide layer. The pass energy of 50 eV was used with a monochromatic Al Kα X-ray source (1486.6 eV). The static and sliding contact angle was measured by Contact Angle System OCA of dataphysics. The water contact angle (CA) of the coating with different layer number (*n*), even (*n* = 6, 10, 14, 18, 22, 26) and odd (*n* = 7, 11, 15, 19, 23, 27), was evaluated. In PSC/TMS-SiO_2_ coating, odd *n* represents the TMS-SiO_2_ surface with hydrophobic property and even *n* is comprised of PSC layer with hydrophilic property. The sliding angles (SAs) of water droplets were examined by adjusting the tilting angle of the coated SS surface placed on the sample stage associated with the CA measuring apparatus having built-in software controlled tilting property. The SAs of the water droplet on coated SS surfaces were accurately measured by tilting the samples from 0° to 50° with the increment of 2° until the droplet rolls off. In addition to SAs, the rolling behavior of the water droplet was continuously captured in a video until the droplet was just started to roll. The CAs and SAs presented here are the average of at least three measured values for each sample. The surface roughness of the PSC/TMS-SiO_2_ coating were measured by Veeco Dektak 150 surface profilometer at nanometer resolution by taking the average of three values measured at different positions of each sample within the error limit of 3–5%. The same surface profilometer was also used to measure the thickness by introducing a step in the coating with the help of a scalpel and the tip of the stylus scanned over it, the step height determines the coating thickness and the data were averaged from three measurements. The digital and microscopic images were obtained to observe the large area surface morphology and cross-cut test of the coating. The optical microscopic images of cross shaped scribe made on the coating by using a scalpel were captured from Carl Zeiss Axio ImagerM2m Microscope System.

The degree of adhesion of PSC/TMS-SiO_2_ coatings were determine by a cross-cut technique and the adhesion rating was assessed with the application of tape on the scribed area, thereby the percentage of coating detached was classified under the range of lowest 0B to upper 5B, in agreement to ASTM D3359–02^56^. For the molecular permeability test, 0.05 mM of PAA was blended with 10 ml NMP with the addition of 0.5 g PSC and placed under stirring for 6–8 h to obtain a homogeneous solution. Simultaneously, 0.1 g of TMS-SiO_2_ was dissolved in 10 ml of toluene to obtain 1% solution and in the same manner solution mixture with 2 to 5% TMS-SiO_2_ was also prepared for comparison. The TMS-SiO_2_ solution was mixed with PSC mixture, followed by stirring of 1 h and the final mixture was cast on the microscopic glass slides and subjected to 60 °C for 8–10 h to evaporate the solvent. Finally, the samples were immersed in deionized water for 10 h to obtain freestanding films and vacuum dried. The barrier ability of PSC/TMS-SiO_2_ cast films was determined through oxygen gas permeability according to ASTM standard E 96 by employing a Yanagimoto Co., Ltd. gas permeability analyzer (GTR-100GW/30X)^56, 74^. Similarly, the water vapor permeability test was performed as reported previously^57, 74^ and the data was presented as an average of three values within the error limit of 3–5%.

The electrochemical impedance spectroscopy (EIS) and polarization tests were conducted with a PGSTAT30 potentiostat of Autolab. The uncoated and coated 316SS act as a working electrode and platinum as a counter electrode. The saturated calomel electrode (SCE) was used as a counter electrode. For electrochemical measurements, the circular region of diameter 1 cm was exposed to 3.5% NaCl solution. The polarization measurements were conducted at starting sweep potential of −0.1 V vs. open circuit potential (OCP) and the sweep rate was 1 mV/s. The EIS were carried out at OCP within a frequency range of 10^5^ to 10^–2^ Hz with the amplitude of 10 mV. The fitting of the electrical equivalent circuits was performed on Nova (version 1.1) software. Cyclic voltammetry was conducted in the same way as other electrochemical tests except the electrolyte solution, i.e., 1 M H_2_SO_4_ solution.

### Electronic supplementary information (ESI) available

FTIR Spectra of PANI, PSC, SiO_2_, TMS-SiO_2_ and schematic representation of silica surface functionalization (Fig. S1). SEM images of SiO_2_ and TMS-SiO_2_ with their respective histogram of sphere diameter, the surface morphology of PSC composite at different magnifications (Fig. S2). TEM images of SiO_2_ and PSC composite (Fig. S3) and thermogravimatric spectra of PANI, PSC, SiO_2_ and TMS-SiO_2_ (Fig. S4). The redox catalytic behavior of PSC/TMS-SiO_2_ coating compared to uncoated samples and the mechanism of transformation in different states of PANI (Fig. S5). Optical microscopic images of cross shaped scribe on PSC/TMS-SiO_2_ coating before and after immersion in 3.5% NaCl for 100 h with 24 hour monitored EIS (Fig. S6). Digital and microscopic images of polished and PSC/TMS-SiO_2_ coated 316SS exposed to 3.5% NaCl solution for 240 h (Fig. S7). The surface wettability parameters and thickness of PSC/TMS-SiO_2_ coating with respect to TMS-SiO_2_ content and layer number (*n*) (Tables S1 and S2), respectively. The influence of pH = 1–5 on surface wettability parameters of PSC/TMS-SiO_2_ coating (Table S3). The self-cleaning behavior of PSC/TMS-SiO_2_ coating at different SAs (Video S1) and the behavior of water droplet at steady SA of 8° (Video S2). The real time rolling behavior of water continuously dropped on PSC/TMS-SiO_2_ coated SS coupon placed on a tilted glass slide (Video S3).

## Electronic supplementary material

Supplementary Information

Video S1

Video S2

Video S3
