# Supplementary material for: Super-hydrophobic multilayer coatings with layer number tuned swapping in surface wettability and redox catalytic anti-corrosion application
Source: Sci Rep. 2017 Jun 30;7:4403. doi: 10.1038/s41598-017-04651-3 (PMC5493639; doi:10.1038/s41598-017-04651-3)
Supplement: Supplementary file 1 — Supplementary Information [file 41598_2017_4651_MOESM1_ESM.doc]

**Electronic Supplementary Information**

**Super-hydrophobic multilayer coatings with layer number tuned swapping in surface wettability and redox catalytic anti-corrosion application**

**Junaid Ali Syed, Shaochun Tang, Xiangkang Meng^*^**

*National Laboratory of Solid State Microstructures, Collaborative Innovation Center of Advanced Microstructures, College of Engineering and Applied Sciences, and Institute of Materials Engineering, Nanjing University, Jiangsu, People’s Republic of China*

_________________________

*To whom correspondence should be addressed. E-mail: mengxk@nju.edu.cn

Tel: (+86) 25 8368 5585. Fax: (+86) 25 8359 5535.

**Figure captions:**

**Fig. S1.** FT-IR Spectra of (a) PANI, (b) PSC, (c) SiO_2_, (d) TMS-SiO_2_ with the magnified peaks, and (e) schematic representation of silica spheres surface functionalization.

**Fig. S2.** The SEM images and histogram of diameters for 60 randomly selected spheres (a, c) silica and (b, d) TMS-SiO_2_. The surface morphology of PSC composite at (e) low and (f) high magnification.

**Fig. S3.** TEM images of (a, b) silica spheres and (c, d) PSC at low and high magnification.

**Fig. S4.** Thermogravimetric spectra of (a) PANI, (b) PSC, (c) SiO_2_ and (d) TMS-SiO_2_.

**Fig. S5.** The redox catalytic behavior of (a) PSC/TMS-SiO_2_ coating and its comparison with uncoated samples, and (b) the proposed mechanism of transformation in different states of PANI.

**Fig. S6.** Optical microscopic images of cross shaped scribe on PSC/TMS-SiO_2_ coating, (a) before and (b) after immersion in 3.5% NaCl for 100 h (c) 24 hourly monitored EIS during 100 h of immersion.

**Fig. S7.** Digital images of (a) uncoated, (b) PSC/TMS-SiO_2_ coated 316SS, (c) uncoated and (d) coated 316SS exposed to 3.5% NaCl solution for 240 h. Magnified optical microscopic images of (e) uncoated and (f) coated 316SS coupons after 240 h of immersion.

**Tables:**

**Table S1.** The surface wettability parameters of PSC/TMS-SiO_2_ coating as a function of TMS-SiO_2_.

**Table S2.** The surface wettability parameters and thickness of PSC/TMS-SiO_2_ coating with respect to layer number (*n*).

**Table S3.** The influence of pH = 1-5 on surface parameters of PSC/TMS-SiO_2_ coating

**Video Legends:**

**Video S1.** The self-cleaning behavior of the PSC/TMS-SiO_2_ coating at different SAs 0° to 6° ± 2°.

**Video S2.** The rolling behavior of water droplet steadied at SA = 8°.

**Video S3.** The real time rolling behavior of water continuously dropped on PSC/TMS-SiO_2_ coated 316SS coupon placed on a glass slide tilted at an angle of 10° ± 3°.


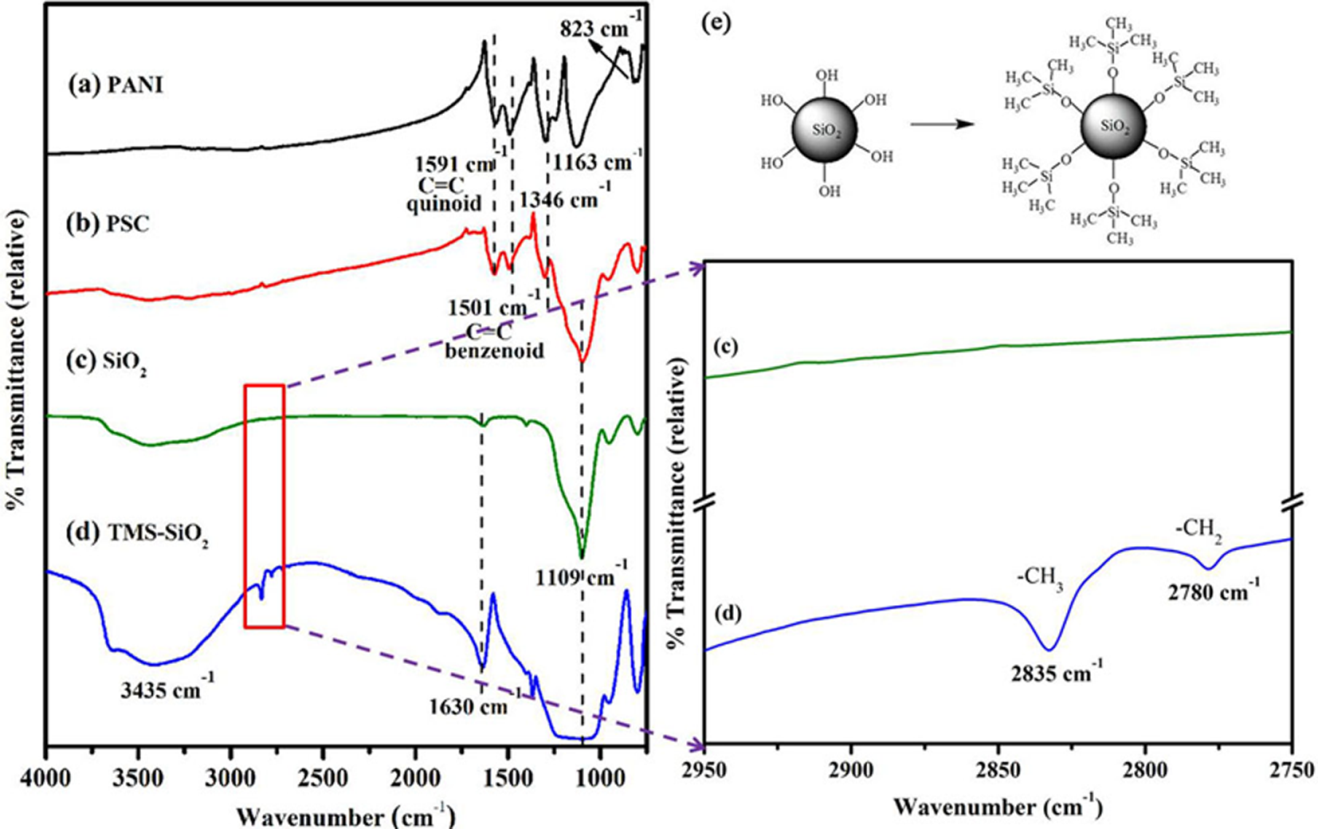


**Fig. S1.** FT-IR Spectra of (a) PANI, (b) PSC, (c) SiO_2_, (d) TMS-SiO_2_ with the magnified peaks, and (e) schematic representation of silica spheres surface functionalization.


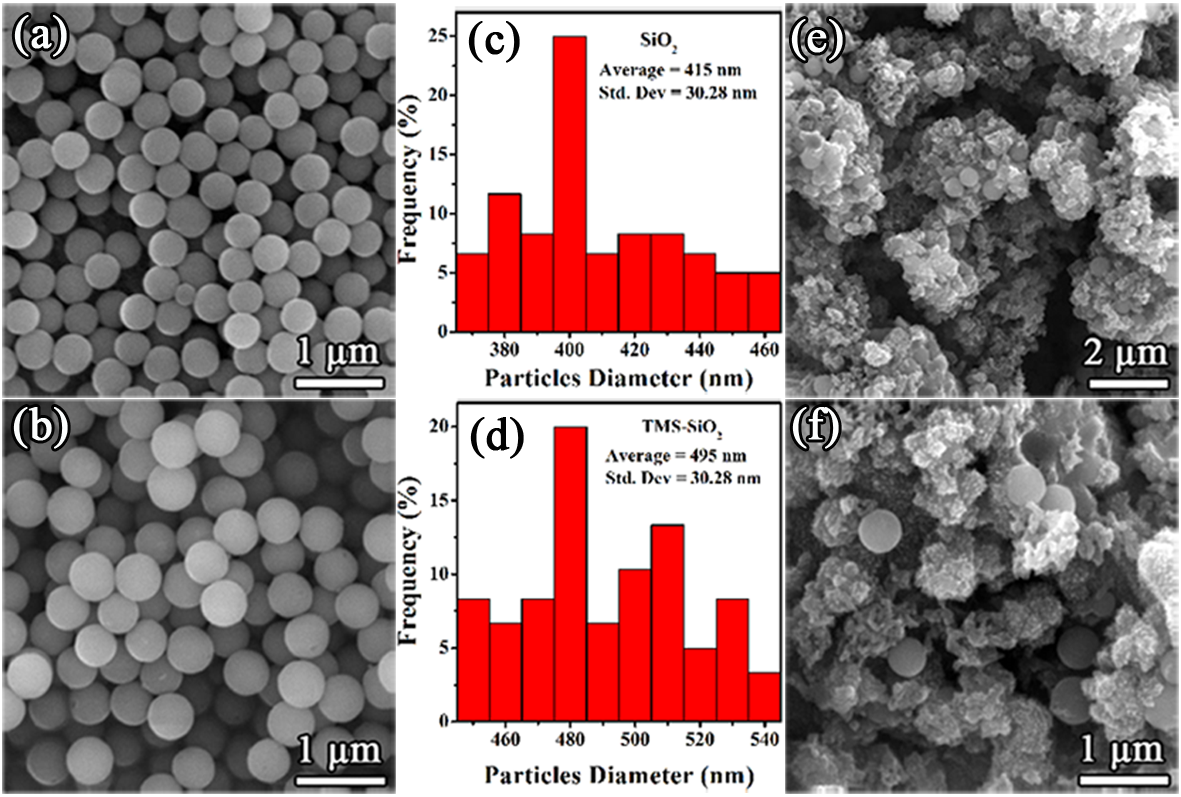


**Fig. S2.** The SEM images and histogram of diameters for 60 randomly selected spheres (a, c) silica and (b, d) TMS-SiO_2_. The surface morphology of PSC composite at (e) low and (f) high magnification.


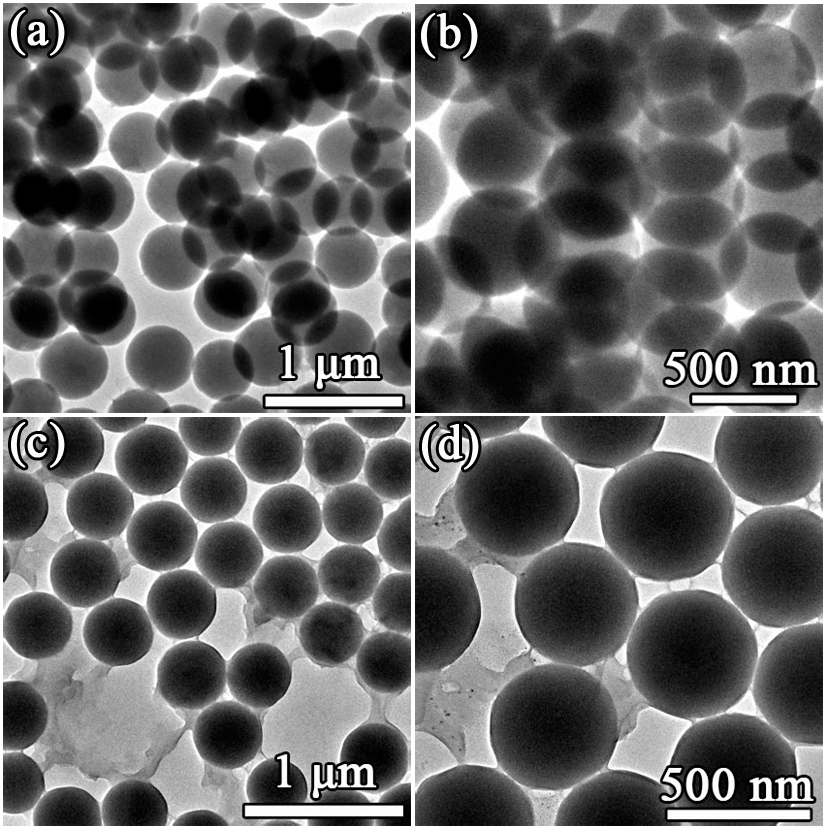


**Fig. S3.** TEM images of (a, b) silica spheres and (c, d) PSC at low and high magnification.


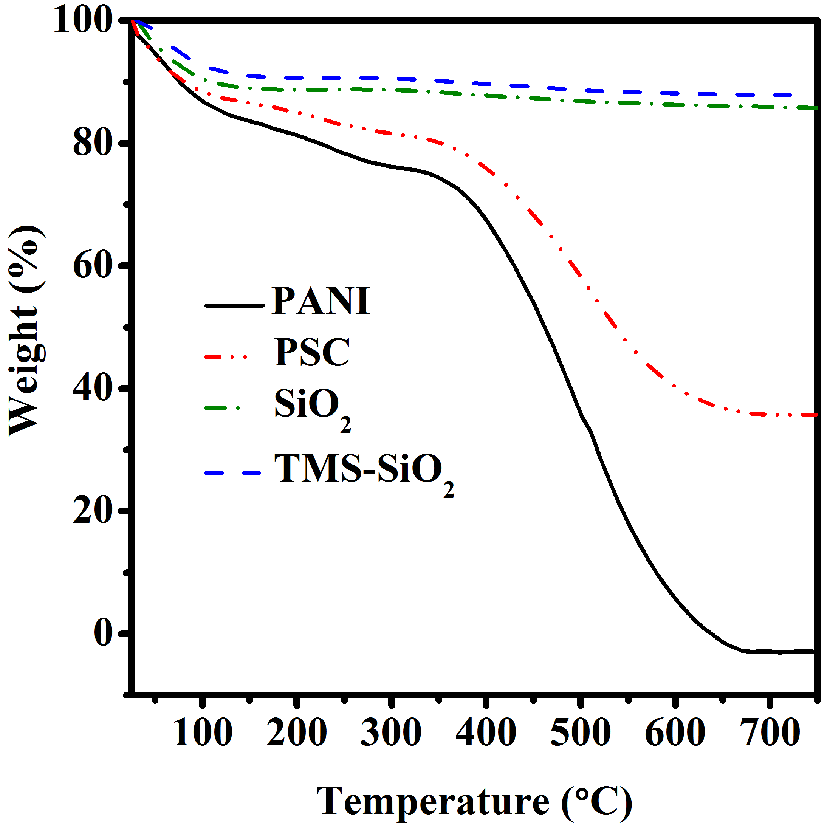


**Fig. S4.** Thermogravimetric spectra of (a) PANI, (b) PSC, (c) SiO_2_ and (d) TMS-SiO_2_.


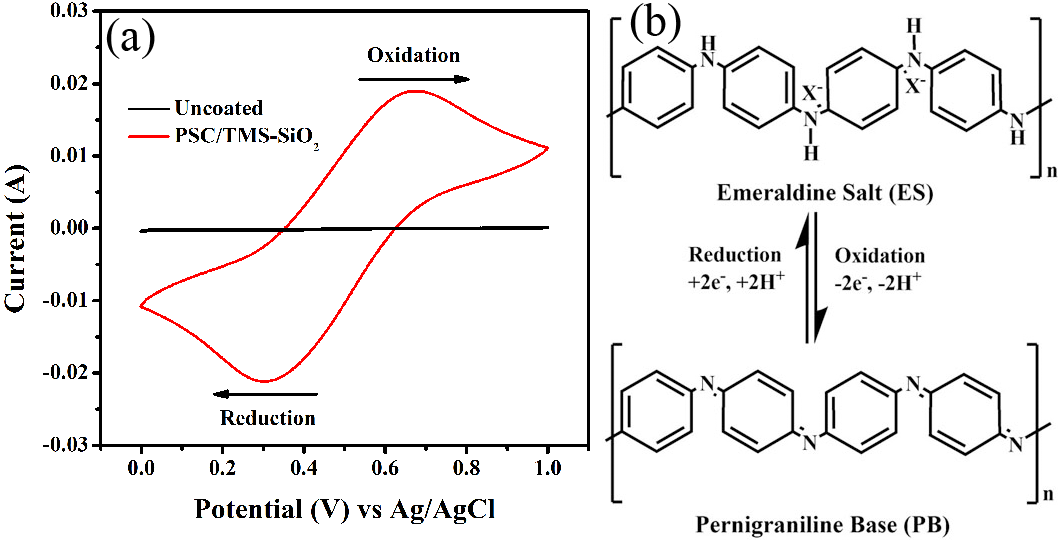


**Fig. S5.** The redox catalytic behavior of (a) PSC/TMS-SiO_2_ coating and its comparison with uncoated samples, and (b) the proposed mechanism of transformation in different states of PANI.


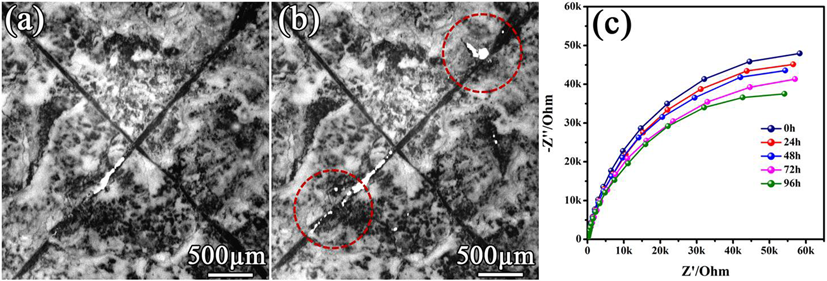


**Fig. S6.** Optical microscopic images of cross shaped scribe on PSC/TMS-SiO_2_ coating, (a) before and (b) after immersion in 3.5% NaCl for 100 h (c) 24 hourly monitored EIS during 100 h of immersion.


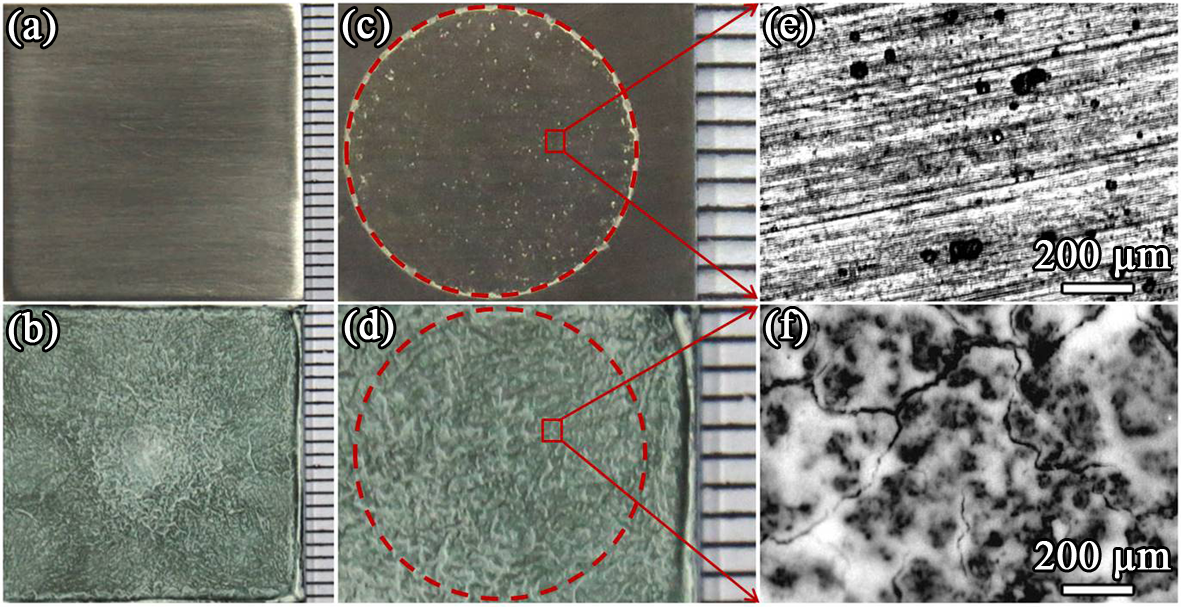


**Fig. S7.** Digital images of (a) uncoated, (b) PSC/TMS-SiO_2_ coated 316SS, (c) uncoated and (d) coated 316SS exposed to 3.5% NaCl solution for 240 h. Magnified optical microscopic images of (e) uncoated and (f) coated 316SS coupons after 240 h of immersion.

**Table S1.** The surface wettability parameters of PSC/TMS-SiO_2_ coating as a function of TMS-SiO_2_.

| TMS-SiO_2_ content | Contact angle (CA) | Slide angle (SA) | Surface roughness (*S*_a_) |
| --- | --- | --- | --- |
| (wt %) | (°) | (°) | (µm) |
| 1 | 125° ± 2.6° | 40° ± 4.2° | 5.19 ± 0.21 |
| 2 | 129° ± 0.88° | 35° ± 3.3° | 6.98 ± 0.19 |
| 3 | 135° ± 2.25° | 23° ± 4.3° | 8.52 ± 0.25 |
| 4 | 142° ± 0.89° | 15° ± 3.1° | 10.13 ± 0.41 |
| 5 | 153° ± 2° | 6° ± 2° | 13.28 ± 0.53 |

**Table S2.** The surface wettability parameters and thickness of PSC/TMS-SiO_2_ coating with respect to layer number (*n*).

| Layer number | Contact angle (CA) | Surface roughness (*S*_a_) | Thickness |  |
| --- | --- | --- | --- | --- |
| (*n*) | (°) | (µm) | (µm) |  |
| 14 | 13.57° ± 1° | 20.17 ± 0.41 | 34.17 ± 3.89 |  |
| 15 | 118° ± 2° | 15.63 ± 0.31 | 37.49 ± 0.81 |  |
| 22 | 45° ± 1° | 13.59 ± 0.27 | 41.59 ± 1.24 |  |
| 23 | 129° ± 3° | 12.12 ± 0.36 | 43.74 ± 2.11 |  |
| 26 | 64° ± 1° | 11.29 ± 0.34 | 58.29 ± 0.74 |  |
| 27 | 136° ± 2° | 10.03 ± 0.42 | 60.03 ± 1.57 |  |

**Table S3.** The influence of pH = 1-5 on surface wettability parameters of PSC/TMS-SiO_2_ coating.

| pH | Contact angle (CA) | Slide angle (SA) | Surface roughness (*S*_a_) |
| --- | --- | --- | --- |
|  | (°) | (°) | (µm) |
| 1 | 110° ± 3.3° | 55° ± 1.65° | 4.28 ± 0.13 |
| 2 | 120° ± 3.6° | 45° ± 1.81° | 5.21 ± 0.16 |
| 3 | 125° ± 2.5° | 38° ± 2.14° | 7.32 ± 0.29 |
| 4 | 140° ± 4.2° | 17° ± 1.68° | 10.42 ± 0.52 |
| 5 | 151° ± 1.5° | 9° ± 0.45° | 11.56 ± 0.46 |
